# Carotenoid aggregates negatively impact chlorophyll levels and disrupt chloroplast development in peaches

**DOI:** 10.1186/s43897-025-00213-8

**Published:** 2026-04-10

**Authors:** Pengfei Wang, Xulei Zhao, Lipeng Liu, Caizhen Gao, Kaijie Zhu, Jiahui Wang, Yan Han, Xueying Zhang, Haijiang Chen, Li Li, Hongbo Cao

**Affiliations:** 1 College of Horticulture, Hebei Agricultural University, Baoding, Hebei 071000 China; 2 National Key Laboratory for Germplasm Innovation & Utilization of Horticultural Crops, College of Horticulture and Forestry Sciences, Huazhong Agricultural University, Wuhan, Hubei 430070 China; 3 Robert W. Holley Center for Agriculture and Health, USA Department of Agriculture-Agricultural Research Service, Cornell University, Ithaca, NY 14853 USA; 4 Plant Breeding and Genetics Section, School of Integrative Plant Science, Cornell University, Ithaca, NY 14853 USA

**Keywords:** Carotenoids, Chlorophyll, Peach (*Prunus persica* L. Batsch), Chromoplast, SGRL

## Abstract

Peach (*Prunus persica* L. Batsch) is among the most economically important fruit tree crops. Carotenoids in peach fruit have been intensively studied because of their relationship with fruit color and nutritional value. High carotenoid accumulation is generally a desirable trait in fruits and is associated with their aggregation in plastids. However, the understanding of the functions of carotenoid aggregates is still limited, especially with respect to their effects on chromoplast differentiation. The present study revealed that carotenoid aggregates have significant biological effects on peach. Lycopene and β-carotene aggregates promoted chlorophyll degradation and thylakoid membrane remodeling, leading to the differentiation of chloroplasts into chromoplasts in peach fruit and callus. However, the phytoene and phytofluene did not produce this effect in peach fruit. Interestingly, during lycopene and β-carotene aggregation, the stay-green-like (*SGRL*) gene was significantly activated, and further confirmed that it was an important factor affecting peach chlorophyll degradation and carotenoid accumulation. Furthermore, molecular dynamics analysis confirmed that lycopene and β-carotene had a greater effect on membrane thickness and rigidity than phytoene. This study contributes to a deeper understanding of peach carotenoid aggregates and their biological effects, confirming the effectiveness of the transgenic peach callus system.

**Supplementary Information:**

The online version contains supplementary material available at 10.1186/s43897-025-00213-8.

## Core

Lycopene and β-carotene aggregates promote chlorophyll degradation in peach fruit and callus, which further leads to thylakoid membrane remodeling and chromoplast differentiation. The *SGRL* gene was identified as an important gene involved in this process. The ‘ZJB’ callus we created is an effective tool for peach molecular biology research because it is easy to stably transform and gene edit.

## Gene and accession numbers

The gene information in this study can be found in the database (https://www.ncbi.nlm.nih.gov/), and the accession numbers are: *PpPSY1* (LOC18783695); *PpPSY2* (LOC18784309); *PpSGRL* (LOC18768837).

## Introduction

Plastids are key organelles with diverse functions in plant cells. The conversion of plastid types is governed by both developmental stages and environmental factors, which are vital for a plant's adaptability and the specialization of its cells (Egea et al. 2010; Llorente et al. 2020). Chloroplasts have been widely studied as photosynthetic plastids. In fully differentiated chloroplasts with mature photosynthetic capacity, chlorophyll, carotenoids, photosystems (including PSI and PSII) and their associated light-harvesting complexes are concentrated in nonstacked and stacked thylakoid domains, respectively (Perez-Boerema et al. 2024). The PSII reaction center complex mainly includes the PsbA (D1), PsbD (D2) and PsbI proteins and the α (PsbE) and β (PsbF) subunits of the Cyt b 559 complex. Photosystem II subunit (LHCII), as a peripheral antenna system, has a multimeric structure composed of six different Lhcb protein subtypes (Lhcb1-6), and each Lhcb monomer precisely coordinates eight chlorophyll a molecules, six chlorophyll b molecules and four carotenoid molecules through noncovalent interactions (Huokko et al. 2021; Ostermeier et al. 2024). Chlorophyll and carotenoids are strategically embedded in the lipid and protein parts of the thylakoid membrane and bind to specific sites of the photosynthetic complex (Rathod et al. 2025).

Among nonphotosynthetic plastids, chromoplasts have also received increasing attention because of their specialized function in carotenoid synthesis and storage (Egea et al. 2010; Li and Yuan 2013). Both leaf senescence and fruit ripening in the natural state require chlorophyll degradation and thylakoid deconstruction (Wei et al. 2023). The degradation of chlorophyll during fruit ripening is thought to be the same as that in leaves, but chloroplast differentiation is not a major event during leaf senescence. Leaf senescence is a morphological change in aged plastids caused by the loss of chlorophyll and does not involve plastid differentiation (Dominguez and Cejudo 2021; Llorente et al. 2020). As the fruit ripens and carotenoids accumulate, chloroplasts differentiate into chromoplasts to accommodate high levels of carotenoids. The conversion of chloroplasts into chromoplasts represents a significant dynamic change in organelles during fruit ripening (Florence and Camara 2006; Ling et al. 2021). Understanding the relationship between carotenoid accumulation and chromoplast development will aid in carotenoid biofortification efforts in plants (Andersen et al. 2021).

Carotenoids and chlorophyll are recognized as the most important plant pigments, and they exist widely in nature and play various functions in plant growth and development (Sun et al. 2018; Wang and Grimm 2021; Yuan et al. 2015). For carotenoid synthesis, phytoene synthase (PSY) is a key rate-controlling step in carotenoid biosynthesis that controls the metabolic flux of carotenoids (Zhou et al. 2022). The overexpression of the *PSY* gene is an effective approach for regulating carotenoid enrichment (Cao et al. 2019). The overexpression of *PSY* significantly increases the carotenoid content in calli. Furthermore, high levels of carotenoids drive the formation of crystalline carotenoids in *Arabidopsis thaliana* and citrus calli, and corresponding changes in plastid morphology are observed (Cao et al. 2012; Maass et al. 2009). *Virus*-mediated expression of *Erwinia herbicola* CrtB (a plant PSY isoenzyme) revealed that it infiltrates green tissues to artificially intervene in the differentiation of chloroplasts into chromoplasts, confirming that carotenoid accumulation is required for the differentiation of chloroplasts into chromoplasts (Llorente et al. 2020). For chlorophyll degradation, stay-green (SGR) is a magnesium-dechelating enzyme that converts chlorophyll a to pheophytin a, which is a key enzyme in the chlorophyll degradation process. In addition, this function of SGR has been shown to be highly conserved from unicellular algae to higher plants (Matsuda et al. 2016). In higher plants, SGR and its homologs can be divided into two subfamilies, namely, the SGR subfamily and the SGR-like (SGRL) subfamily. SGR forms a dynamic multiprotein complex with the LHCII for chlorophyll degradation during aging, accelerating the disassembly of the photosynthetic system (Sakuraba et al. 2015).

Peach is one of several crops of economic importance in the genus *Prunus* and is particularly favored by consumers because of its rich nutritional value and delicious taste. The color of peach pulp can be divided into two types, namely, yellow and white. Yellow-fleshed peach fruit are rich in carotenoids, which greatly increase the nutritional and commodity value of the peach fruit (Cao et al. 2017; Veerappan et al. 2021). The transcription of carotenoid synthesis genes is active during the ripening period of white-flesh peach fruit. The strong transcription of the carotenoid cleavage dioxygenase 4 (*CCD4*) gene leads to the degradation of carotenoids (Brandi et al. 2011). Carotenoid synthesis and storage structures in peach fruits with different flesh colors clearly differ. The plastoglobules of yellow peach fruits are significantly larger than those of white peach fruits (Han et al. 2020). However, the underlying mechanisms regulating carotenoid synthesis and plastid development in peach fruits are still unclear.

The lack of natural carotenoid mutants, except for yellow flesh in peach fruit, combined with recalcitrance to regeneration in tissue culture for transformation makes determining the mechanisms of carotenoid synthesis and storage in peach fruit difficult (Ricci et al. 2020). Carotenoid enzyme inhibitors achieve targeted alterations in carotenoid composition, which is highly valuable for research on carotenoids. The CPTA [2-(4-chlorophenylthio)-triethylamine hydrochloride] lycopene cyclase (LCYB) inhibitor has been shown to induce lycopene accumulation (Lu et al. 2019). The phytoene desaturase (PDS) inhibitor NFZ induces photobleaching in leaves and leads to the accumulation of phytoene and phytofluene (Lu et al. 2019). Because mature peach fruit has a strong capacity for carotenoid synthesis, the use of inhibitors significantly affects the content and composition of carotenoids (Lado et al. 2016). Moreover, the callus is a useful model for studying carotenoid metabolism, and callus transformation is highly important for understanding the mechanism of carotenoid metabolism (Li and Yuan 2013; Yuan et al. 2015). The citrus carotenoid engineered cell model (ECM) shows that callus cells overexpressing *CrtB* primarily accumulate carotenoid components in the form of β-carotene and convert amyloplasts into crystalline chromoplasts (Cao et al. 2012).

The present study explored the formation of carotenoid aggregates during carotenoid storage and their biological effects. Different carotenoid aggregate forms had different biological effects on chromoplast differentiation. The aggregates of lycopene and β-carotene promoted chlorophyll degradation and thylakoid membrane remodeling, thus initiating the chromoplast differentiation process, whereas phytoene and phytoene aggregates did not induce chromoplast differentiation. In addition, molecular dynamics (MD) analysis revealed that thylakoid membrane stiffness and thickness were easily disrupted by lycopene and β-carotene aggregates but not by phytoene aggregates.

## Results

### CPTA-induced lycopene accumulation promotes chlorophyll degradation in peach fruit

**Fig. 1:**
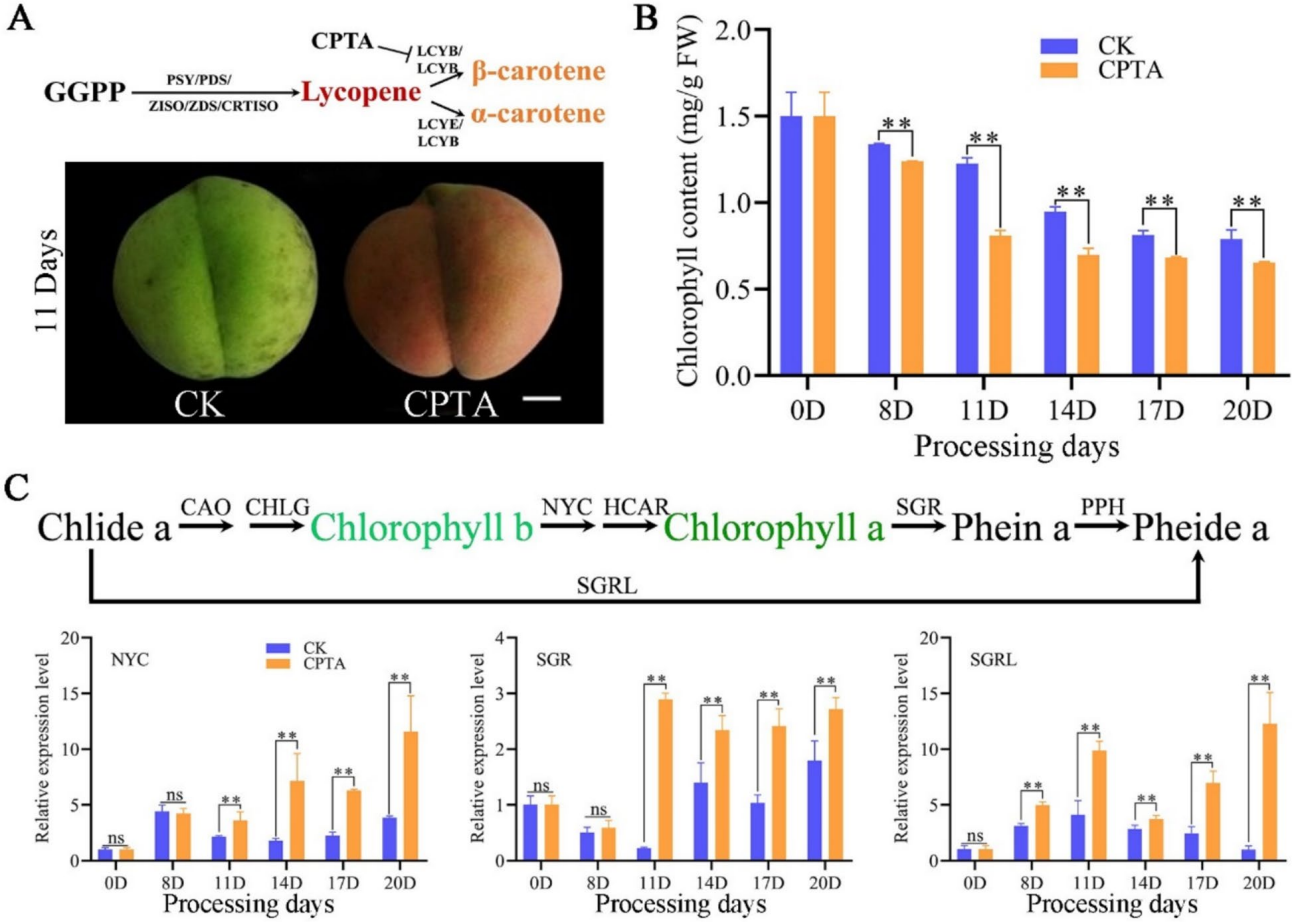
Lycopene accumulation induced by CPTA promotes chlorophyll degradation. **A** The action site of CPTA inhibitor and the observation of peach fruit peel color on the 11th day of treatment (scale bar, 1 cm). NOTE: PSY, phytoene synthase; PDS, phytoene desaturase; ZISO, δ-carotene isomerase; ZDS, δ-carotene desaturase; CRTISO, carotene isomerase; LCYE, lycopene ε-cyclase; LCYB, lycopene β-cyclase. **B** Chlorophyll content of fruit peel at different treatment stages. FW, fresh weight. ** Indicates a significant difference at *P* < 0.01. **C** Relative expression levels of key genes involved in chlorophyll degradation in inhibitor-treated and control peels. NOTE: CAO, Chlorophyllide a oxygenase; CHLG, Chlorophyll synthase; NYC, chlorophyll b reductase; HCAR, 7-Hydroxymethyl chlorophyll a reductase; PPH, Pheophytin pheophorbide hydrolase

The CPTA lycopene cyclase inhibitor inhibited LCYB activity, resulting in lycopene accumulation (Fig. 1A). To assess the effectiveness of CPTA on lycopene accumulation in ripe peach fruit, mature peach fruit from DXMT peaches were soaked in CPTA solution. CPTA treatment resulted in the conversion of green fruit to red fruit after 8 days (Fig. 1A). Analysis of the chlorophyll content revealed that both the CPTA-treated group and the mock control group presented a decreasing trend, but the chlorophyll content in the peel of the CPTA-treated group was significantly lower than that in the control group (Fig. 1B). HPLC-PAD analysis revealed that the peel of the CPTA-treated group contained high concentrations of lycopene, whereas no lycopene was detected in the peel of the control group (Table 1). The contents of phytoene and phytofluene also significantly increased in the CPTA treatment group, whereas the content of β-carotene decreased (Table 1). However, lycopene accumulation and chlorophyll content changes were not observed when immature (six-ripe stage) peach fruits were treated with CPTA. Because carotenoid biosynthesis accompanied fruit ripening and a relatively low carotenoid synthesis capacity was present in immature fruits (Fig. S2), CPTA did not induce high levels of lycopene accumulation in immature fruits. These findings suggested that the excessive accumulation of lycopene induced by CPTA is the main cause of chlorophyll changes rather than the influence of the chemical properties of CPTA. qRT‒PCR analysis revealed that the expression levels of chlorophyll degradation genes, namely, *SGR*, *SGRL*, and chlorophyll b reductase (*NYC*)*,* in the CPTA-treated group were greater than those in the control group (Fig. 1C).

**Table 1:** Carotenoid contents and components in peach fruits (μg/g FW)

Carotenoids	0D	8D		11D		14D		17D		20D	
		CK	CPTA	CK	CPTA	CK	CPTA	CK	CPTA	CK	CPTA
Lycopene	—	—	15.96 ± 0.75**	—	18.91 ± 0.23**	—	31.91 ± 2.06**	—	37.06 ± 0.79**	—	33.76 ± 0.61**
β-carotene	3.27 ± 0.25	3.2 ± 0.04**	1.26 ± 0.05	3.14 ± 0.19**	0.66 ± 0.05	2.92 ± 0.05**	0.62 ± 0.03	1.95 ± 0.05**	0.26 ± 0.02	1.36 ± 0.09**	0.27 ± 0.03
phytoene	0.19 ± 0.01	0.3 ± 0.01	0.89 ± 0.08**	0.4 ± 0.03	1.44 ± 0.07**	0.41 ± 0.02	2.31 ± 0.05**	0.50 ± 0.02	4.81 ± 0.62**	0.56 ± 0.03	2.65 ± 0.2**
phytofluene a	0.14 ± 0.01	0.21 ± 0.01	0.72 ± 0.06**	0.29 ± 0.01	1.04 ± 0.02**	0.3 ± 0.01	1.60 ± 0.09**	0.35 ± 0.01	4.24 ± 0.15**	0.32 ± 0.01	1.9 ± 0.08**
phytofluene b	0.13 ± 0.01	0.15 ± 0.01	0.23 ± 0.05	0.14 ± 0.01	0.32 ± 0.01**	0.14 ± 0.01	0.47 ± 0.02**	0.15 ± 0.01	0.51 ± 0.02**	0.27 ± 0.01	0.37 ± 0.01**

“—” Indicates that no detection

*FW* Fresh weight

^**^ Indicates a significant difference at *P* < 0.01 between CK and CPTA

### Lycopene aggregates drive chromoplast formation and suppress photosynthesis-related gene expression in peach fruit

**Fig. 2:**
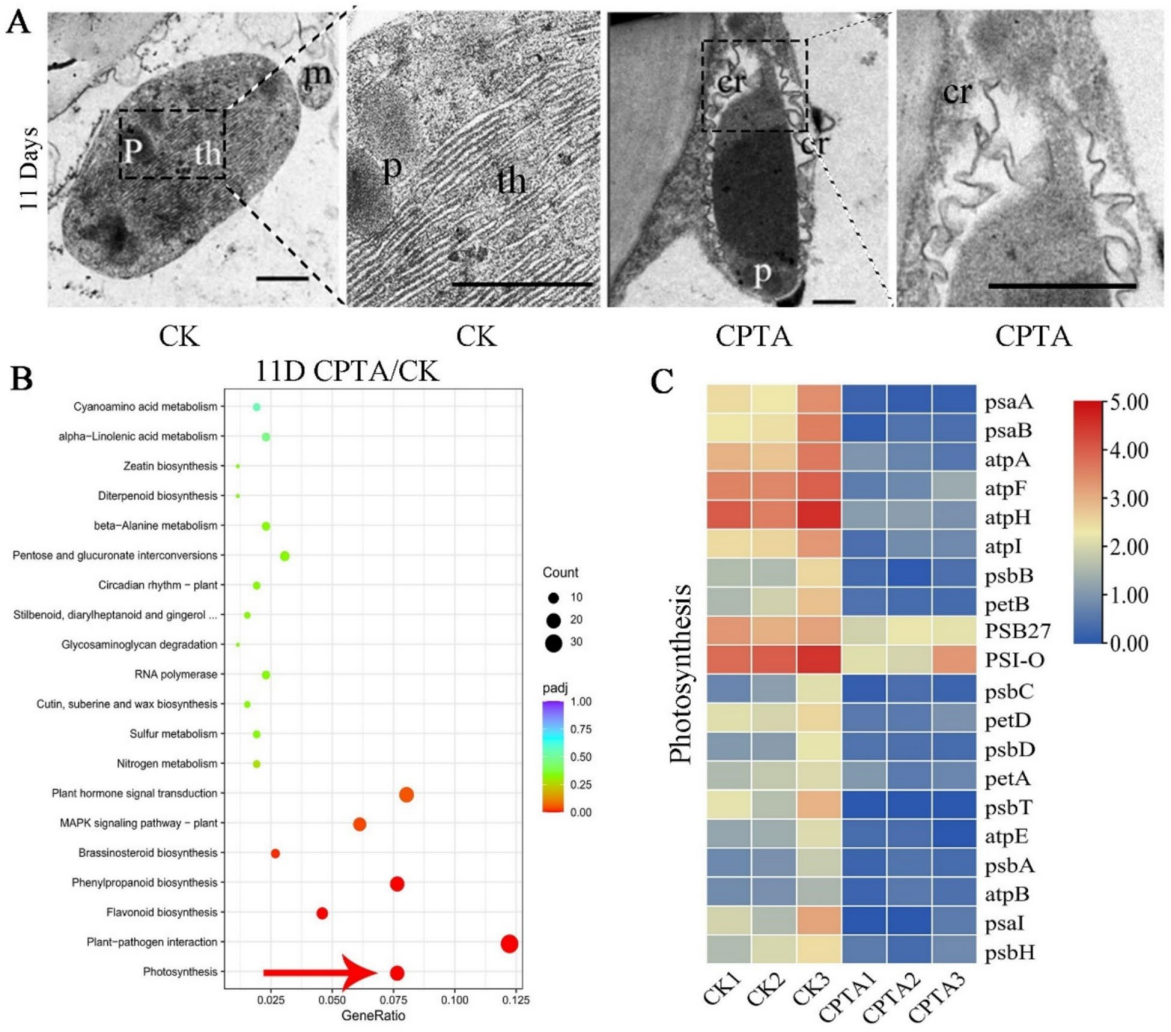
Microstructural observation and transcriptome analysis. **A** Electron microscopic observation results of CPTA-treated and control fruit peels on the 11th day. NOTE: p, plastoglobule; cr, crystalline; th, thylakoid; m, mitochondria (scale bar, 0.5 μm). **B** KEGG enrichment map of Day 11 samples from the CPTA treatment and control groups. **C** Photosynthetic pathway gene expression profiles of Day 11 CPTA-treated and control samples

Carotenoid accumulation is often accompanied by the development of chromoplasts and the formation of special carotenoid storage substructures (Li and Yuan 2013; Sun et al. 2018). The cells of the DXMT peach peel displayed chloroplast-like structures, and a few plastoglobules (PGs) were observed around the stacked thylakoids (Fig. 2A). After CPTA treatment, chloroplast-to-chromoplast conversion was observed in the peel, resulting in the disappearance of thylakoid structures and the formation of crystalline lycopene aggregates (Fig. 2A).

To gain a deeper understanding of the impact of carotenoid aggregation on fruit, transcriptomic analysis was performed on the Day 8 and Day 11 samples, resulting in 650 million high-quality reads from 15 RNA-Seq libraries, which were mapped to the peach genome (Table S2). PCA of the samples revealed a clear separation of the different treatments and a strong correlation of the biological replicates (Fig. S3). The transcript levels of nine DEGs associated with the chlorophyll metabolism and carotenoid metabolism pathways were analyzed via qRT‒PCR to verify the reliability of the RNA-Seq data (Fig. S4). Statistical analysis of the DEG expression data revealed 857 DEGs (532 downregulated and 325 upregulated) in the Day 8 samples and 1337 DEGs (469 downregulated and 868 upregulated) in the Day 11 samples, resulting in a total of 2194 DEGs in the two periods (Fig. S5A). KEGG analysis was used to enrich 249 shared differential genes from both periods (Fig. S5B), among which 2 DEGs were involved in photosynthesis and 1 DEG was involved in carotenoid metabolism (Fig. S5C). The KEGG results of the DEGs identified in the Day 11 samples revealed significant enrichment of the photosynthetic pathway (Fig. 2B), and the expression of all the DEGs associated with the photosynthetic pathway in the CPTA treatment group tended to decrease (Fig. 2C).

### β-Carotene aggregates promote chlorophyll degradation in peach calli, accompanied by chromoplast differentiation

**Fig. 3:**
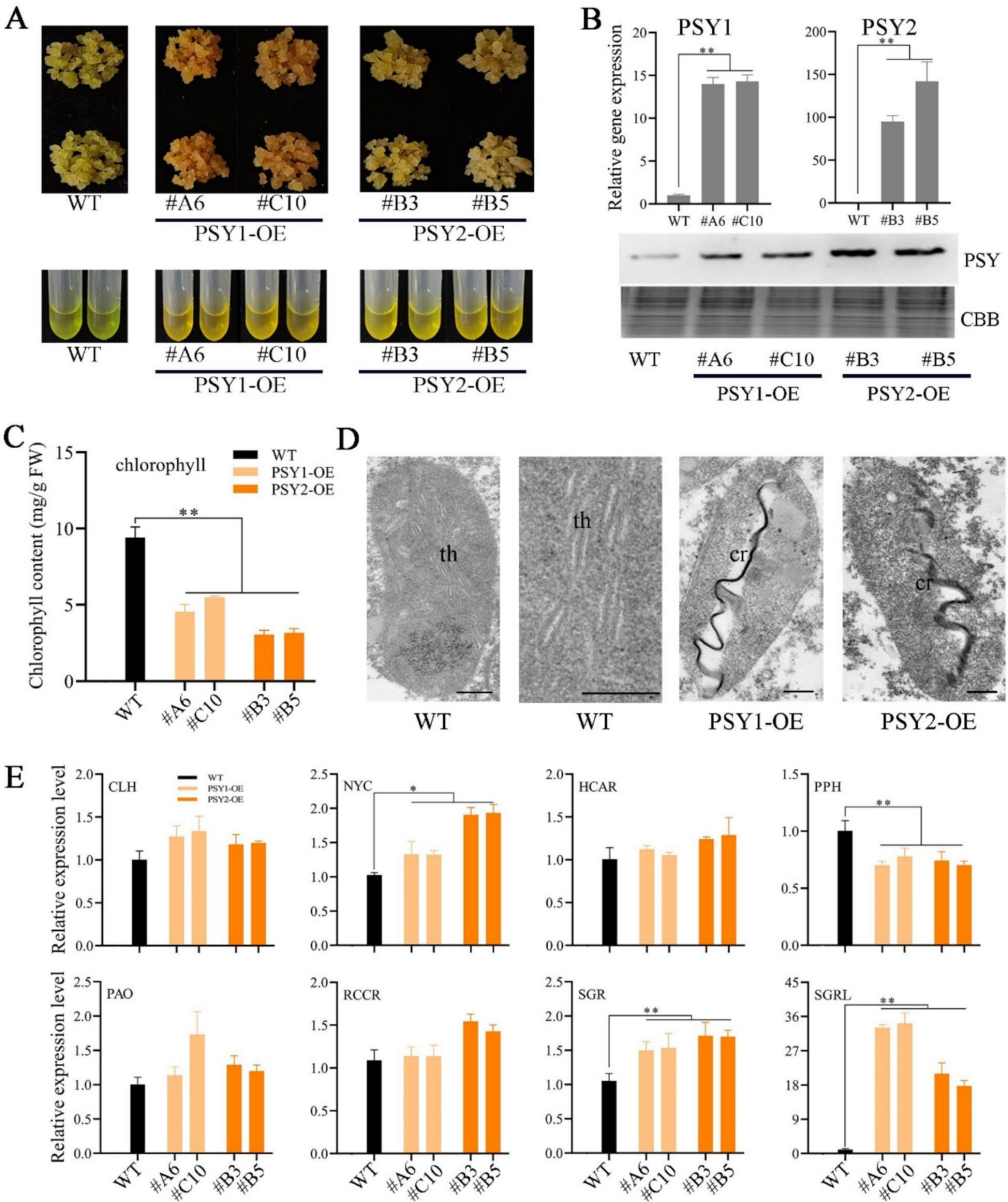
β-Carotene promotes chromoplast differentiation in peach calli. **A** Observation on the phenotypes and color of pigment extracts of different PSY transgenic callus lines. **B** Relative expression levels of *PSY1* and *PSY2* in the overexpressing callus lines as determined via qRT‒PCR. Western blot analysis of PSY protein levels in different *PSY* transgenic callus lines. Coomassie brilliant blue (CBB) staining was used as a loading control. ** Indicates a significant difference at *P* < 0.01. **C** Analysis of the chlorophyll content in different *PSY* transgenic callus lines. FW, fresh weight. **D** Electron microscopic observation of different *PSY* transgenic callus lines (scale bar, 0.5 μm). **E** Relative expression levels of chlorophyll degradation pathway genes in different *PSY* transgenic callus lines as determined via qRT‒PCR. NOTE: CLH, Chlorophyllase; NYC, chlorophyll b reductase; HCAR, 7-Hydroxymethyl chlorophyll a reductase; PPH, Pheophytin pheophorbide hydrolase; PAO, Pheophorbide a monooxygenase; RCCR, Red chl catabolite reductase; SGR, Stay-green; SGRL, Stay-green-like. * and ** indicate significant differences at *P* < 0.05 and *P* < 0.01, respectively

To further confirm the role of carotenoid aggregates in inducing chlorophyll degradation and plastid differentiation, β-carotene aggregation was induced in peach calli. Calli are frequently utilized as a model to study the metabolism of carotenoids (Cao et al. 2012). In the present study, one green callus line (‘ZJB’) induced from peach anthers provided good material for studying chloroplast differentiation, as it presented high chlorophyll accumulation and contained chloroplast-like structures (Fig. 3A). The 35S promoter-controlled *PpPSY*-overexpressing callus systems (*PSY1*-OE and *PSY2*-OE) were successfully established via *Agrobacterium*-mediated transformation via ‘ZJB’ calli. The transgenic calli were yellow or orange in color with carotenoid-rich characteristics (Fig. 3A). Both the gene expression and protein levels of PSY were significantly increased in the overexpressing callus lines (Fig. 3B). Compared with those in the wild type, the levels of phytoene and β-carotene were significantly greater in the transgenic calli (Table 2), whereas the chlorophyll content was significantly lower (Fig. 3C). TEM analysis revealed that the plastids in the wild-type ‘ZJB’ callus were mainly chloroplast-like, whereas the *PpPSY* transgenic calli contained chromoplasts (Fig. 3D). The expression levels of genes involved in the chlorophyll degradation pathway were next examined. qRT‒PCR analysis revealed that the expression levels of most genes in the ‘ZJB’ wild-type and transgenic lines were similar, except for significantly upregulated transcript levels of *NYC*, *SGR,* and *SGRL*. In particular, *SGRL* was upregulated 17~34-fold in the *PSY* lines (Fig. 3E).

**Table 2:** Carotenoid contents and components in the transgenic callus lines (μg/g FW)

Carotenoids	ZJB	PSY1#A6	PSY1#C10	PSY2#B3	PSY2#B5
Phytoene	—	0.236 ± 0.019**	0.208 ± 0.029**	0.206 ± 0.013**	0.199 ± 0.002**
Lutein	0.93 ± 0.001**	—	—	—	—
α-cryptoxanthin	—	0.084 ± 0.001**	0.081 ± 0.003**	0.075 ± 0.003**	0.068 ± 0.001**
β-cryptoxanthin	0.071 ± 0.001	0.115 ± 0.009	0.075 ± 0.004	0.079 ± 0.003	0.076 ± 0.003
α-carotene	0.094 ± 0.003	0.215 ± 0.019**	0.118 ± 0.005	0.111 ± 0.003	0.106 ± 0.001
β-carotene	0.353 ± 0.017	3.558 ± 0.182**	2.676 ± 0.157**	1.543 ± 0.036**	1.432 ± 0.007**

“—” Indicates that no detection

*FW* Fresh weight

^**^Indicates a significant difference at *P* < 0.01 between the ‘ZJB’ and PSY transgenic lines

### Phytoene and phytofluene aggregation have no significant effect on chlorophyll in peach fruit

**Fig. 4:**
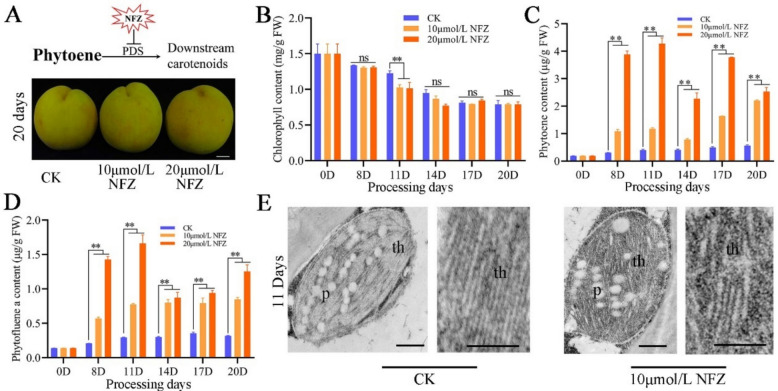
Phytoene and phytofluene accumulation induced by NFZ does not lead to chlorophyll degradation, thylakoid deconstruction, or chromoplast differentiation. **A** The action site of NFZ inhibitor and the observation of fruit peel color on the 20th day of treatment (scale bar, 1 cm). **B** Chlorophyll content of fruit peel at different stages after NFZ treatment. ** Indicates a significant difference at *P* < 0.01. **C** Phytoene content of fruit peel at different stages after NFZ treatment. ** Indicates a significant difference at *P* < 0.01. **D** Phytofluene content of fruit peel at different stages after NFZ treatment. F: Electron microscopic observation results of NFZ-treated and control fruit peels on Day 11. NOTE: p, plastoglobule; th, thylakoid (scale bar, 0.5 μm)

In addition to the most significant accumulation of lycopene and β-carotene, the changes in phytoene and phytofluene contents were also significant in the CPTA-treated group and the calli overexpressing *PSY* (Tables 1 and 2). To determine whether phytoene and phytofluene aggregates play important roles in chlorophyll degradation and chromoplast differentiation, DXMT peaches were permeabilized with NFZ to study the effects of phytoene and phytofluene on chromoplast differentiation. Compared with that of the control group, peach fruit color did not change after NFZ treatment (Fig. 4A), and the chlorophyll content of the peel did not significantly differ, with differences occurring only on Day 11 (Fig. 4B). The phytoene and phytofluene contents increased significantly (Fig. 4C-D). However, an intact chloroplast structure was found after NFZ treatment (Fig. 4E), indicating that the accumulation of phytoene and phytofluene did not induce thylakoid deconstruction and chromoplast differentiation. These results indicated that different carotenoid components have different effects on plastid development.

### SGRL participates in carotenoid aggregation-induced chlorophyll degradation and is involved in the regulation of carotenoids

The *SGRL* chlorophyll-degrading gene was significantly activated in both CPTA-treated fruit and *PSY*-overexpressing peach calli (Figs. 1C and 3E), which suggested that *SGRL* may play an important role in the response to carotenoid accumulation. The peach genome contains *SGR* and *SGRL* genes (International Peach Genome et al., 2013). Phylogenetic analysis of SGR and SGRL revealed that SGR and SGRL belong to two subfamilies (Fig. S6A). Both SGR and SGRL contain the SGR domain and C-terminal domain (Fig. S6B). Previous studies have shown that SGR plays an important regulatory role in the metabolism of chlorophyll and carotenoids. For example, SGR degrades chlorophyll and interacts with PSY to negatively regulate carotenoid synthesis (Luo et al. 2013; Zhu et al. 2021). However, the role of SGRL in pigment synthesis remains unknown.

**Fig. 5:**
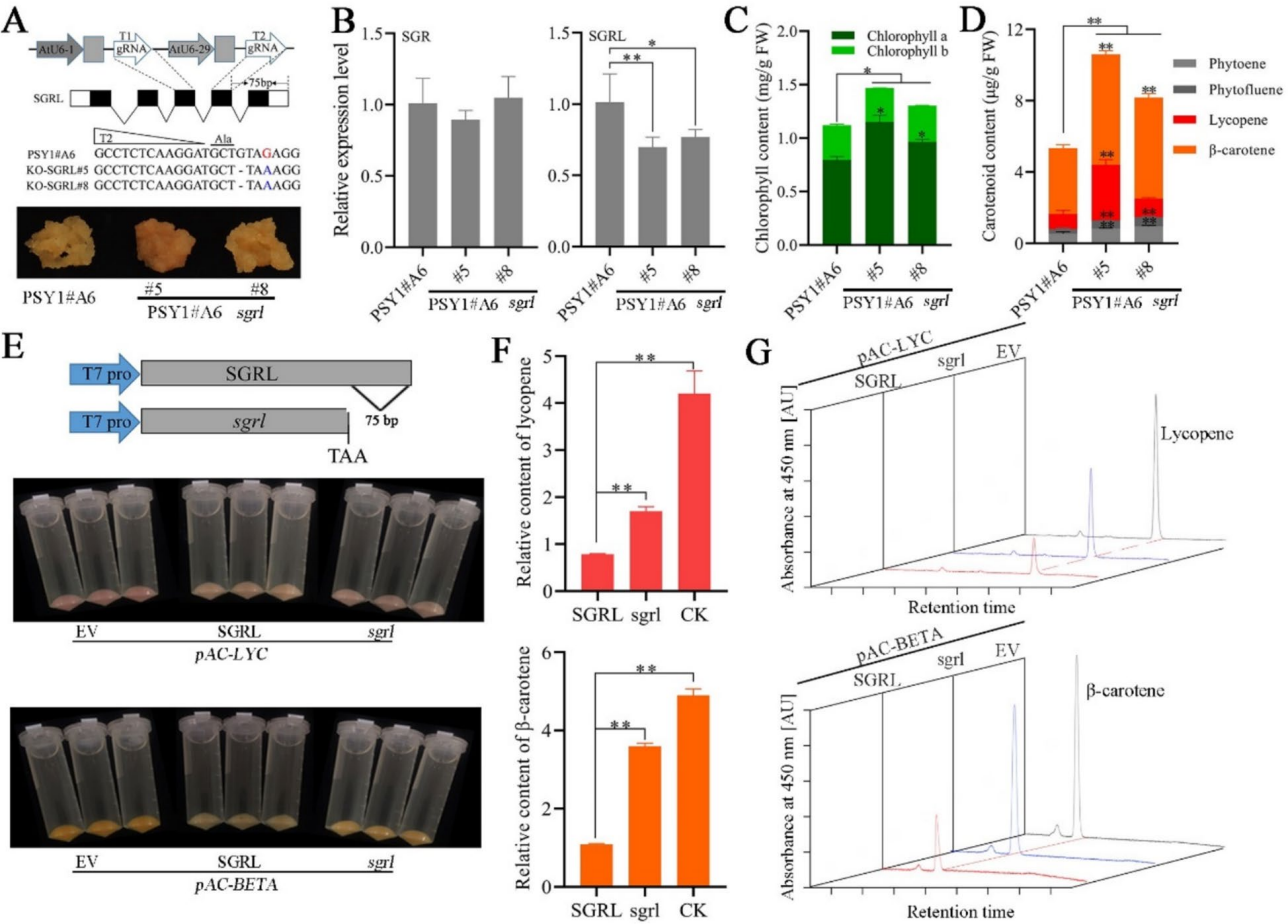
SGRL regulates chlorophyll and carotenoid metabolism. **A** *sgrl* CRISPR/Cas9 knockout. The binary construct contained two target sites for the *sgrl* exon. Gene editing sites and callus phenotypes of two *sgrl-ko* lines. The names of the transgenic lines represent the overexpression of *PSY1* (PSY1#A6) and the two mutant lines with SGRL knockout based on PSY1#A6 (PSY1#A6 *sgrl*#5 and #8). **B** Relative expression levels of *SGRL* and homologous gene *SGR* in 25-day-old calli. * and ** indicate significant differences at *P* < 0.05 and *P* < 0.01, respectively. **C** Chlorophyll content of 25-day-old calli tissues of different transgenic lines. * Indicates a significant difference at *P* < 0.05. **D** Carotenoid content of 25-day-old calli tissues of different transgenic lines. ** Indicates a significant difference at *P* < 0.01. **E** Color of carotenoid engineered bacteria. The upper part shows the phenotype of engineered bacteria overexpressing EV (empty vector), SGRL, mutant *sgrl* to pAC-LYC plasmid (lycopene). The lower part shows the phenotype of engineered bacteria overexpressing EV (empty vector), SGRL, mutant *sgrl* to pAC-BETA plasmid (β-carotene). **F** Expressed as the lycopene and β-carotene contents in the engineered bacteria (EV, SGRL and mutant *sgrl*), respectively. ** Indicates a significant difference at *P* < 0.01. **G** Chromatograms of carotenoids in the engineered bacteria (EV, SGRL and mutant *sgrl*) detected by high performance liquid chromatography (HPLC), respectively

The *SGRL* gene was knocked out in peach calli overexpressing *PSY1* through CRISPR gene editing. The two selected lines (#5 and #8) had nucleotide deletions, and protein translation terminated prematurely (Fig. 5A). Compared with that of the *PSY1* transgenic line, the color of the *sgrl-ko* lines was darker. In addition, the expression level of *SGRL* was significantly reduced in the *sgrl-ko* lines, whereas the expression of *SGR* was not affected (Fig. 5B). Pigment analysis revealed that the chlorophyll content (especially the chlorophyll a content) significantly increased in the *sgrl-ko* line (Fig. 5C). Notably, the carotenoid content was significantly greater in the *sgrl-ko* line than in the transgenic line overexpressing *PSY1* alone (Fig. 5D). Therefore, we speculated that the C-terminal domain of SGRL may be potentially important for PSY function. The effects of *SGRL* and its mutant *sgrl* on carotenoid synthesis were tested via the use of a carotenoid-engineered bacterial system. When the complete *SGRL* sequence and mutant *sgrl* were introduced into *E. coli* to produce lycopene or β-carotene, respectively, the color of the carotenoid-engineered bacterial transformants containing *SGRL* became significantly lighter, whereas the color of the transformants containing *sgrl* was restored (Fig. 5E). HPLC-PAD further confirmed the effects of *SGRL* and *sgrl* on carotenoid synthesis (Fig. 5F-G). Based on the results of gene-edited *sgrl-ko* lines and carotenoid engineering bacteria experiments, we confirmed that intact SGRL has the function of inhibiting carotenoid synthesis, while the *sgrl* mutant weakened this effect. Subsequently, we performed yeast two-hybrid interaction verification and found that the full-length SGRL interacted with PSY (CrtB) in peach, and the C-terminal domain of SGRL was essential for maintaining the interaction between SGRL and PSY (CrtB) (Fig. S7). In addition, we used alphafold3 to perform molecular docking of SGRL and mutant *sgrl* with PSY and found that the C-terminal structure of SGRL occupied the substrate channel when PSY performed its function, while the mutant *sgrl* could not affect the activity of PSY due to the disappearance of the interaction. These results indicated that SGRL plays an important regulatory role in photosynthetic pigment metabolism and that the 75-bp nucleotide sequence encoded by *SGRL* is a key domain for regulating carotenoid synthesis ability, thereby providing an important target for subsequent carotenoid biofortification.

### Proteomic analysis reveals complex changes in plastid proteins during carotenoid aggregation

**Fig. 6:**
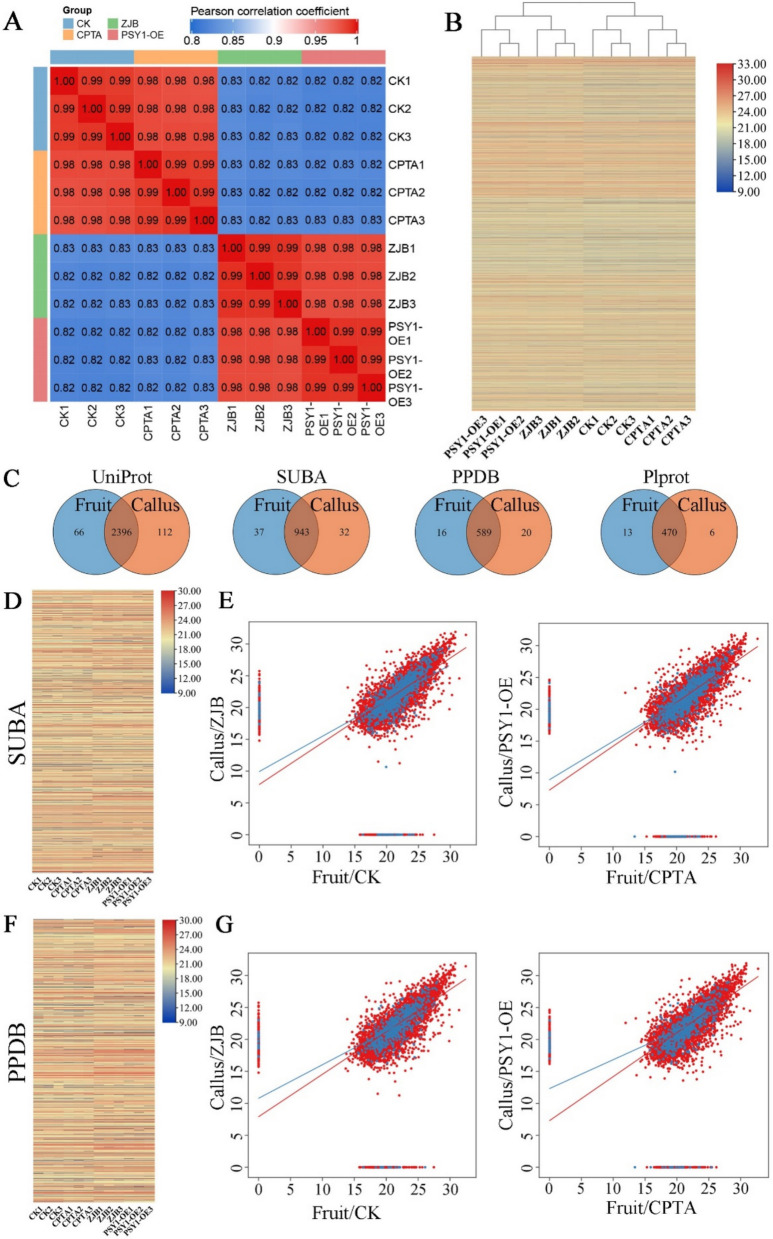
Comparative analysis of proteomics in peach fruit and callus. **A** Pearson correlation coefficient between different samples (fruit and callus). **B** Fruit and callus protein expression profiles. **C** Venn diagram of plastid proteins in different protein databases of fruit and callus. **D** Plastid protein expression profile of the SUBA database. **E** Linear regression fitting analysis of plastid and nonplastid protein expression data in SUBA database. **F** Plastid protein expression profile of the PPDB database. **G** Linear regression fitting analysis of plastid and nonplastid protein expression data in PPDB database

The differentiation of chloroplasts into chromoplasts requires substantial proteome reconfiguration (Barsan et al. 2012). To better understand how carotenoid aggregates influence plastid differentiation at the protein level, proteome detection was used. On the basis of 4D label-free quantitative proteome technology, the whole proteomes of peach peels after 11 days of treatment and *PSY1*-overexpressing calli were identified, resulting in the detection of 7648 proteins (Fig. S8A). PCA was used to assess the degree of sample dispersion and data reproducibility (Fig. S8B). Pearson's correlation coefficient revealed a significant positive correlation between fruit and callus proteins, with a correlation coefficient greater than 0.8 (Fig. 6A). The heatmap also revealed that the fruit and callus protein expression profiles were similar (Fig. 6B), which confirmed the effectiveness of using the callus system to verify fruit carotenoid synthesis and storage. With a focus on the composition of fruit and callus plastid proteins, the plastid proteins of fruits and calli were compared by searching three plastid protein databases (SUBA, PPDB, and PlProt) and the UniProt protein database simultaneously. Plastid proteins in fruits and calli presented more than 97% similarity (Fig. 6C). Furthermore, the plastid protein expression profile and linear regression results confirmed the high similarity and significant positive linear distribution of plastid proteins in fruits and calli (Fig. 6D-G). To evaluate the protein differences between fruits and calli, specific proteins in fruits and calli were analyzed, but effective subclassification statistics could not be performed for these proteins (Table S3). These proteins may play potential roles in the development of fruits or calli.

**Fig. 7:**
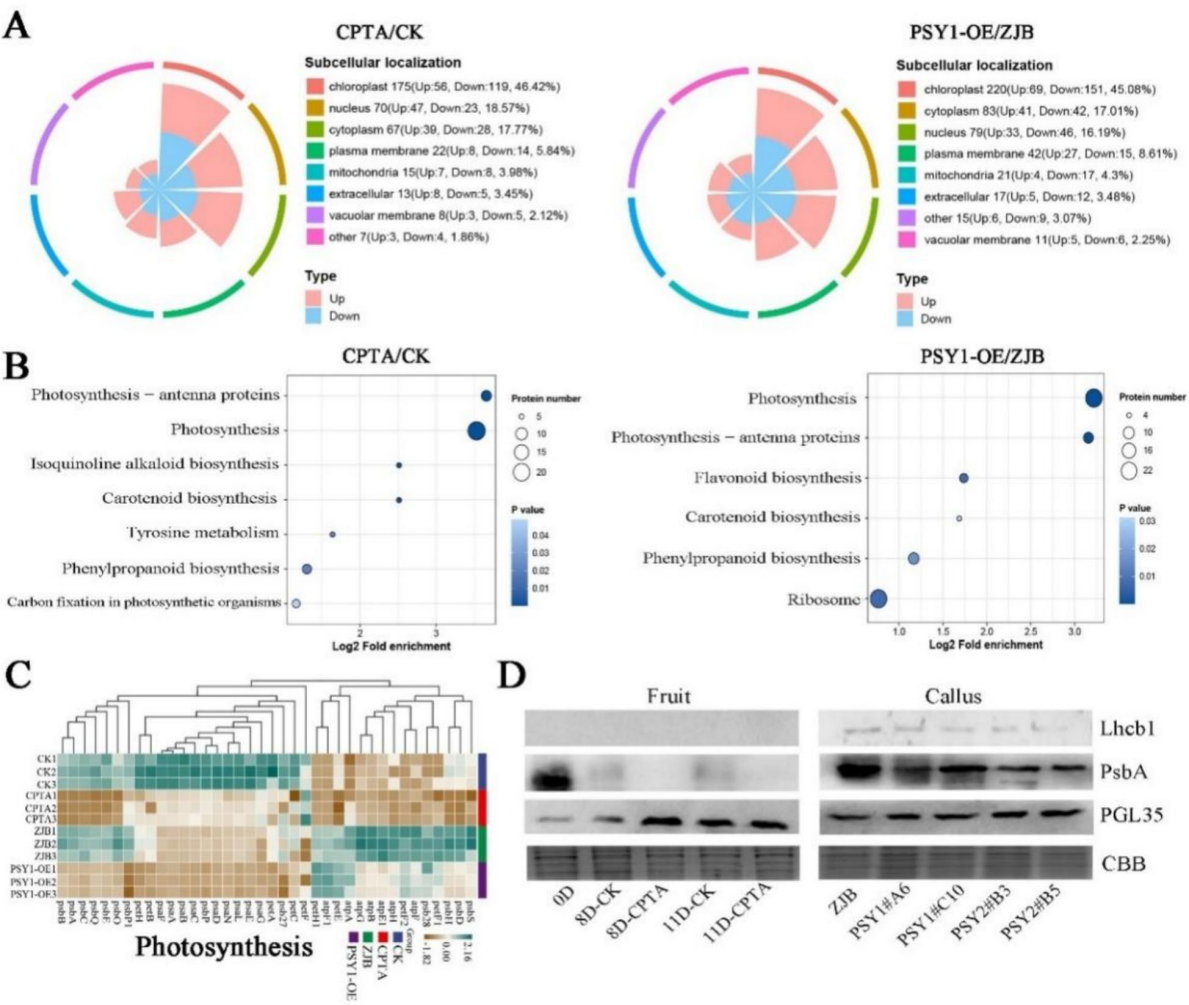
Lycopene and β-carotene aggregates induce profound changes in plastid proteins. **A** DEPs subcellular localization distribution in CPTA/CK and PSY1-OE/ZJB. **B** KEGG enrichment pathway of DEPs in CPTA/CK and PSY1-OE/ZJB. **C** CPTA/CK and PSY1-OE/ZJB photosynthetic system protein expression profiles. **D** Western blot analysis of Lhcb1, PsbA, and PGL35 protein levels in the peach peels of CPTA-treated (Day 8 and Day 11) and different transgenic callus lines. Coomassie brilliant blue (CBB) staining was used as a loading control

In addition, differentially expressed protein (DEP) analysis was performed. In the peel, 377 DEPs were identified, including 171 upregulated proteins and 206 downregulated proteins. Similarly, 488 DEPs were identified in calli, including 190 upregulated proteins and 298 downregulated proteins (Fig. S8C). The prediction of the subcellular localization of DEPs revealed a relatively high percentage of protein in chloroplasts (46.42% in peel and 45.08% in callus) (Fig. 7A), suggesting complex changes in plastid proteins during chromoplast differentiation. Moreover, the enriched KEGG pathways of the DEPs between the peel and callus samples were highly consistent and included mainly photosynthesis, photosynthesis-antenna proteins, and carotenoid biosynthesis (Fig. 7B). Among the GO-enriched cell component categories, the number of thylakoid-related proteins tended to decrease in both the CPTA-treated group and the transgenic callus line (Fig. S9). Consistent with the results of the microscopic observations, chromoplast differentiation induced by lycopene and β-carotene was accompanied by significant changes in the structure of the plastid inner membrane system (Figs. 2A and 3D). Furthermore, the expression of photosynthesis proteins in the CPTA-treated peels and transgenic calli was downregulated (Fig. 7C and Fig. S10). Photosystem II and its surrounding binding proteins are important light-harvesting complexes, and the expression levels of two core proteins (Lhcb1 and PsbA) are important. Lhcb1 was not detected in the peel, and the PsbA protein level in the peel of plants subjected to CPTA treatment was lower than that in the control. In addition, the Lhcb1 and PsbA protein expression levels in the PSY callus were lower than those in the wild type (Fig. 7D). Western blot analysis of chromoplast proteins (PGL35) revealed higher levels in the CPTA-treated group and *PSY* transgenic callus line than in the control group, indicating the completion of chromoplast differentiation.

### Carotenoid aggregates have different aggregation effects on lipid bilayers

**Fig. 8:**
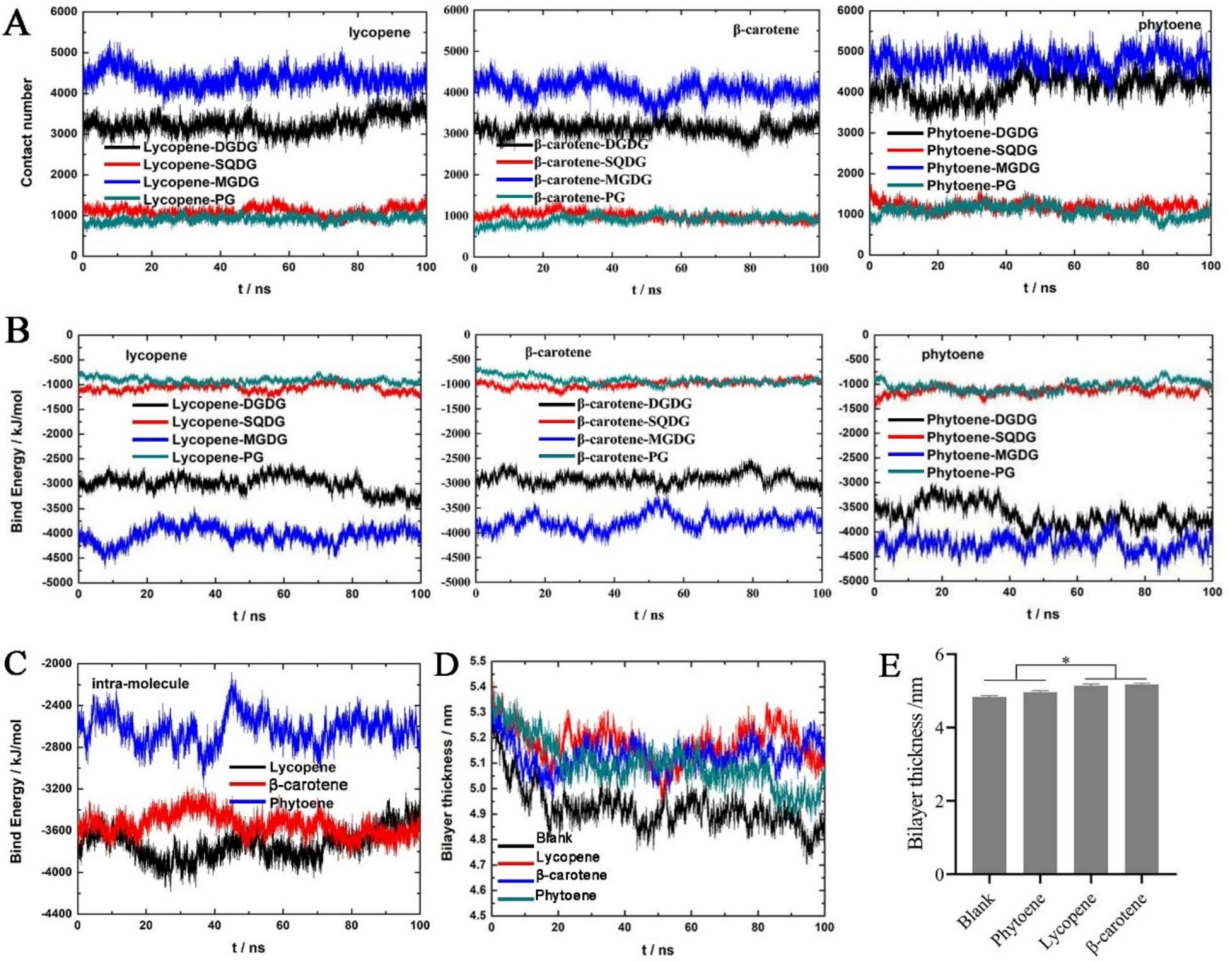
Molecular dynamics simulation analysis of the aggregation of different carotenoid components in lipid bilayers. **A** Analysis of the changes in the number of contacts between different carotenoid molecules and lipid molecules during the simulation process. **B** Analysis of the changes in interaction energy between different carotenoid molecules and lipid molecules during the simulation process. ‘-’ represents attraction, and the absolute value of the number represents the interaction energy. **C** Analysis of the change in interaction energy between molecules of the same carotenoid during the simulation process. ‘-’ represents attraction, and the absolute value of the number represents the interaction energy. **D** Effect of carotenoid aggregates on lipid bilayer thickness during simulations. **E** Quantification of membrane thickness by different carotenoid aggregates in the last 10 ns. * Indicates a significant difference at *P* < 0.05

Significant changes in plastid proteomics indicated the positive effects of lycopene and β-carotene on chromoplast differentiation, which caused remodeling of the plastid endomembrane system to adapt to different carotenoid aggregates. Because carotenoids tend to be embedded in thylakoid membrane phospholipid bilayers (Popova and Andreeva 2013) and to form aggregates when their content is high, the carotenoid aggregates affecting the phospholipid bilayer may be the driving force that induces adaptation of the plastid membrane system. To confirm the effects of lycopene and β-carotene aggregation on membrane systems and to determine why phytoene had no significant effect, molecular dynamics (MD) simulations were performed. MD simulation is an excellent tool for evaluating the overall impact of carotenoids on membrane structure (van Eerden et al. 2015). Carotenoids perform their functions mainly by being embedded in the lipid bilayer of membrane systems, similar to cholesterol (Mostofian et al. 2020). To evaluate the effect of carotenoids on the membrane structure, MD simulations with a duration of 100 ns were performed at 1 bar and 310 K. The model of the thylakoid membrane was based on experimental characterization, with four lipids (PG(16:1(3t)), DGDG(18:3(9,12,15)), MGDG(18:3(9,12,15)), and SQDG(18:3(9,12,15)) at a ratio of 15:30:40:15 and carotenoid molecules (β-carotene, lycopene, and phytoene), corresponding to molar carotenoid concentrations of 10% in the thylakoid membrane simulation system. Snapshots of the system at different moments were simulated according to the dynamic trajectory of each system (Fig. S11). Carotenoids, as photoprotective pigments, are embedded in lipid bilayers through intermolecular forces and are involved in the regulation of lipid membrane fluidity, which may depend on the structure of the carotenoid molecule itself. The MD results revealed that the interaction energies between the lycopene and β-carotene molecules confirmed that they formed firmer aggregates in the lipid bilayers (Fig. 8A-B and Table 3), which may be responsible for the formation of crystalline carotenoids from lycopene and β-carotene. However, phytoene and lipid molecules had more contact numbers and interaction energies (Fig. 8A-B and Table 3). The intermolecular force of carotenoids showed that the intermolecular attraction of lycopene and β-carotene was greater than that of phytoene (Fig. 8C). In addition, lycopene and β-carotene had greater effects on the thickness of lipid membranes than did phytoene (Fig. 8D-E). In summary, the results indicated that lycopene and β-carotene have greater aggregation effects than phytoene does and that lycopene and β-carotene crystalline carotenoids affect the stability and rigidity of lipid membranes.

**Table 3:** Carotenoid and lipid intermolecular contact numbers and interaction energies

Lipid molecules	Contact number			Interaction energy (kJ/mol)		
	lycopene	β-carotene	phytoene	lycopene	β-carotene	phytoene
DGDG	3552	3210	4287	−3279.42	−3001.81	−3796.00
SQDG	1209	891	1155	−1156.16	−902.62	−1124.31
MGDG	4289	4018	4749	−3961.06	−3725.71	−4222.51
PG	967	925	956	−955.94	−933.19	−948.90
Total	10,017	9044	11,147	−9352.58	−8563.32	−10,091.73

## Discussion

Chlorophyll degradation and carotenoid accumulation are essential processes for fruit ripening in many horticultural plants. During fruit ripening and senescence, color changes are accompanied by changes in chloroplast morphology and structure (Sierra et al. 2023; Yuan et al. 2015). Carotenoid accumulation is known to promote the formation of chromoplasts, but owing to their large variety, whether and how different types of carotenoid aggregates play important roles in controlling chromoplast differentiation are unclear. The accumulation of carotenoids is the result of a combination of synthesis, degradation, and storage (Li and Yuan 2013). Owing to the degradation ability of CCD4, mature white-fleshed peach fruit contains trace amounts of carotenoids but maintains a strong carotenoid synthesis capacity (Brandi et al. 2011), thus providing convenience for exploring the specific components of carotenoids involved in the formation of chromoplasts. The present study demonstrated that different types of carotenoid aggregates have different effects on plastid development. Specifically, lycopene and β-carotene aggregates activate the SGRL-regulated chlorophyll degradation process, deconstruct the thylakoids, and induce chromoplast formation. However, phytoene and phytofluene aggregates do not induce chromoplast differentiation.

### Specific carotenoid aggregates induce chromoplast differentiation

The development and structural changes of chromoplasts are generally recognized as important biological processes for carotenoid sequestration in plants (Fraser et al. 2007). There are different types of chromoplasts (globular, tubular, crystalline, fibrous, and membranous), and these types are associated with differences in carotenoid content and composition (Choi et al. 2021; Li et al. 2016). Tomato and watermelon fruits are enriched mainly in lycopene (crystalline). Carrots accumulate α-/β-carotene (crystalline), and marigold flowers are enriched in lutein (tubular) (Egea et al. 2010; Li and Yuan 2013). Although recent studies have shown that carotenoid accumulation promotes chromoplast formation, how different carotenoid aggregates affect the development of chromoplasts remains elusive (Li et al. 2016; Sun et al. 2022a). The model of chloroplast differentiation into chromoplasts in leaves involves two overlapping stages, where excessive accumulation of phytoene competes for binding to the chlorophyll‒carotenoid photosynthetic protein complex and interferes with photosynthesis (Llorente et al. 2020). The accumulation of downstream carotenoids may be the direct cause of chromoplast differentiation (Llorente et al. 2020). The present study demonstrated that the downstream carotenoids lycopene and β-carotene induce the differentiation process of chloroplasts into chromoplasts (Figs. 2A and 3D) and that phytoene and phytofluene do not eliminate chloroplast properties (Fig. 7E). Plastoglobules are derived from thylakoid membranes, which adapt to changing environmental conditions and development through lipid remodeling (Austin et al. 2006; Bykowski et al. 2021). In chloroplasts, chlorophyll and carotenoids are present in the lipid and protein components of thylakoid membranes, but differences in carotenoid components affect thylakoid morphology and membrane structure (Bykowski et al. 2021; Nogueira et al. 2013). Plastids have multiple abilities to adapt to changes in carotenoid content, and carotenoid properties and contents trigger different regulatory mechanisms (Austin et al. 2006). For example, high levels of phytoene are detected in plastoglobules, whereas the accumulation of lycopene requires thylakoid membranes to regulate the MGDG content to improve their adaptability (Austin et al. 2006; Goss and Latowski 2020). The increased levels of β-carotene and lycopene crystal structures in CrtB + CrtI tomato fruits alter the properties of the thylakoids and grana (Nogueira et al. 2013). The present study demonstrated that the aggregation of lycopene and β-carotene leads to disorganization of the thylakoid membrane structure and the formation of a new membrane structure that accommodates the crystal-structured carotenoids (Figs. 2A and 3D).

### Chlorophyll degradation is involved in the differentiation of chloroplasts into chromoplasts

Chlorophyll and carotenoids are important photosynthetic pigments for living organisms (Sun et al. 2023). In chloroplasts, chlorophyll is responsible for absorbing light energy to complete the energy conversion process; carotenoids are used as auxiliary light-harvesting pigments for photoprotection (Murchie and Lawson 2013). The degreening process caused by chlorophyll degradation is a normal phenomenon associated with leaf senescence and fruit ripening (Wei et al. 2023). Chlorophyll and carotenoids in the green tissue of plants (leaves or immature fruits) may have a metabolic equilibrium point that maintains normal plant growth, as there is a mutual feedback mechanism between chlorophyll and carotenoids (Gong et al. 2024). Studies have shown that chromoplasts are generated from preexisting chloroplasts during green fruit maturation and that chromoplast differentiation involves major changes in carotenoid and chlorophyll contents (Egea et al. 2010; Rodiger et al. 2021). The present study demonstrated that SGRL-induced chlorophyll degradation in peach is one of multiple metabolic processes involved in carotenoid aggregate-induced plastid differentiation (Figs. 1C and 3E). Previous studies have shown that SGR degrades chlorophyll in the chlorophyll‒protein complex and is involved in regulating the degradation of the photosynthetic system in green plants. Moreover, the chlorophyll degradation process occurs before the degradation of photosynthetic proteins (Shimoda et al. 2016). In tomato, SGRL interacts with pheophytin pheophorbide hydrolase (SlPPH) and light-harvesting complex a2 (SlLHCa2) to promote chlorophyll degradation (Yang et al. 2020). This study used gene editing technology, carotenoid engineering bacteria, and yeast two-hybrid interaction analysis to prove that SGRL has a negative regulatory effect on carotenoid synthesis, and the carboxyl terminus of SGRL is essential for maintaining the interaction between PSY and SGRL. Protein molecular docking results suggest that the carboxyl terminus of SGRL affects the substrate capture ability of PSY during GGPS-PSY interaction, which has reference value for subsequent carotenoid biofortification engineering (Fig. 4). In tomato, the interaction between SGR and PSY inhibits fruit carotenoid synthesis, demonstrating the regulatory role of SGR in carotenoid metabolism (Luo et al. 2013). CsSGR inhibits carotenoid synthesis in citrus calli by directly interacting with CsPSY1 (Zhu et al. 2021).

### Different carotenoid components have different aggregation effects

In the functional chloroplasts of fully developed plants, carotenoids are present in photosynthetic protein complexes in the thylakoid membrane structure, and changes in their content are closely related to the lipid remodeling process that occurs in the thylakoid membrane system (Bykowski et al. 2021). Specifically, carotenoid molecules that are not bound to photosynthetic complexes are freely dispersed in the lipid bilayer and can cause changes in membrane rigidity and stability through van der Waals interactions with phospholipids (Domonkos et al. 2013). In different types of carotenoid mutants, changes in the lutein/carotene ratio are related to the thylakoid grana, thylakoids, and photosystem PSII supercomplex in chloroplasts, further affecting the fluidity of the thylakoid membrane (Bykowski et al. 2021). Previous results have confirmed that differences in carotenoid content affect the structural and physical properties of lipid bilayers (Mostofian et al. 2020). The present findings indicated that high contents of lycopene or β-carotene prevent the presence of thylakoid membranes, whereas phytoene and phytofluene do not affect the thylakoid membrane structure (Figs. 2A, 3D and 7E). In the *pds*-silenced tobacco leaves, the granum stacking of thylakoids in the variegated parts is normal compared with that in the control. The concentration of the thylakoid membrane proteins in the *pds*-silenced plants significantly increased at the same chlorophyll content (Wang et al. 2009).

MD simulations also revealed that lycopene and β-carotene have strong intermolecular forces, whereas phytoene has strong forces on lipid bilayers, suggesting that lycopene and β-carotene are prone to form aggregates in lipid bilayers and that the aggregation of lycopene and β-carotene leads to thickening of the lipid membranes (Table 3 and Fig. 8). The photoprotective effect of carotenoids generally shields the photosynthetic system from damage caused by singlet oxygen by reducing the fluidity of the thylakoid membrane (Adamkiewicz et al. 2013). The interaction between carotenoid molecules (especially aggregate structures) and lipid molecules determines the formation and physical properties of lipid membrane structures (Adamkiewicz et al. 2013). The head groups of polar carotenoids (lutein and zeaxanthin) are decisive for the orientation of the carotenoids in the lipid bilayer, acting as rivets and decreasing the fluidity of the polar regions of the membrane; thus, they increase membrane rigidity, whereas nonpolar carotenoids (lycopene and β-carotene) are located inside the hydrophobic core of the lipid bilayer (Augustynska et al. 2015; Socaciu et al. 2002). X-ray diffraction data have indicated that lycopene and β-carotene have greater disruptive effects on the membrane structure compared to lutein and zeaxanthin (McNulty et al. 2008). The presence of 2.5 mol% lycopene in the lipid bilayer induces structural loosening of the acyl chain in dipalmitoyl phosphatidylcholine membranes (Suwalsky et al. 2002). In addition, EPR spin label and anisotropy studies have confirmed the membrane-disordering effects of β-carotene and lycopene, respectively (Socaciu et al. 2000; Strzałka and Gruszecki 1994). These findings indicate that carotenoid aggregation has a negative effect on the formation and stability of photosynthetic membrane systems, especially when lycopene and β-carotene aggregate to form crystal structures. We hypothesize that after a certain solubility limit, lycopene or β-carotene will precipitate out of the lipid bilayer and produce carotenoid crystals, which will damage the photosynthetic membrane and complete the differentiation process of chloroplasts into chromoplasts.

**Fig. 9:**
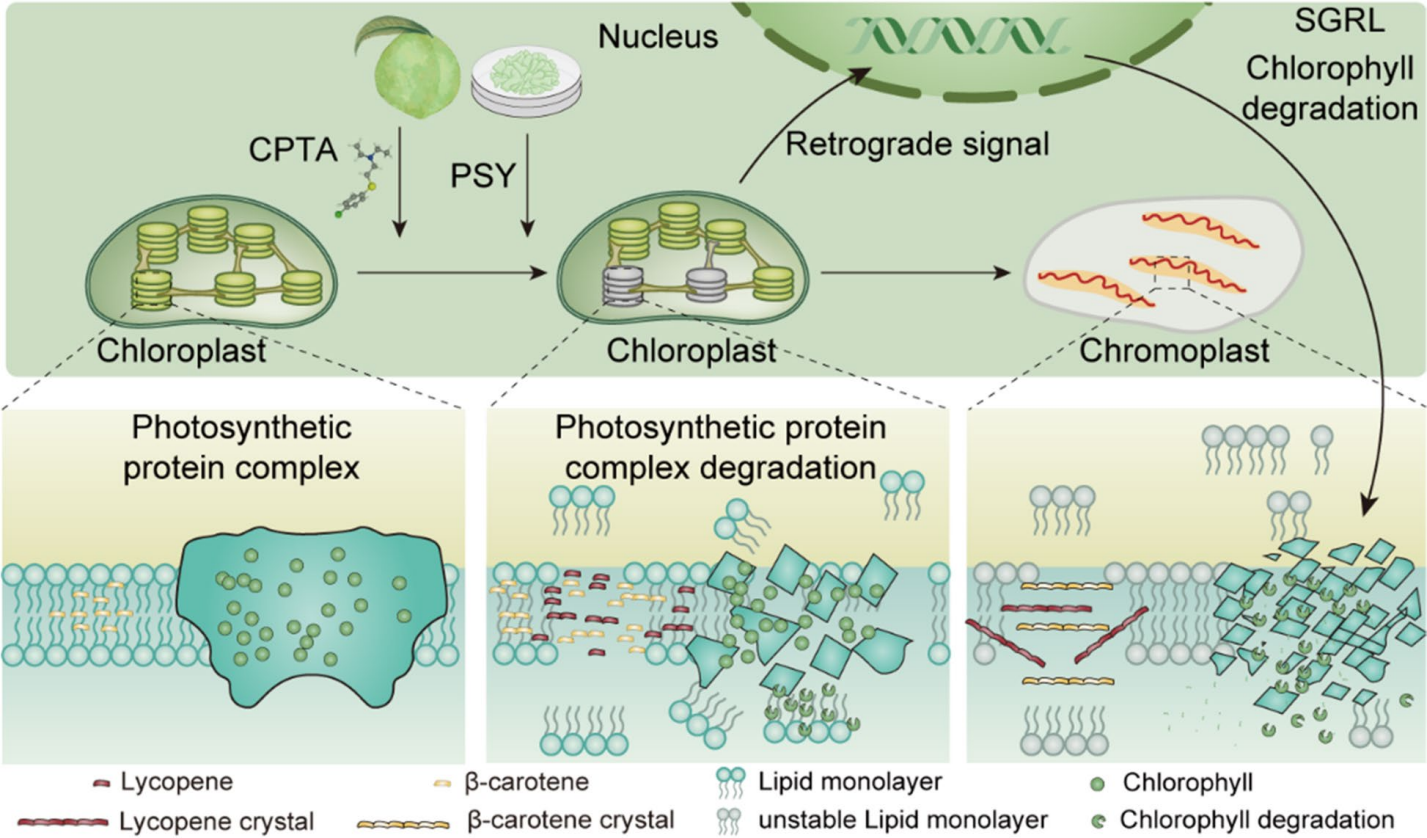
Model for chromoplast differentiation and chlorophyll degradation induced by carotenoid aggregates

In conclusion, this study investigated the effects of different carotenoid aggregates on chlorophyll degradation and chromoplast differentiation in peach fruits and calli. The results of pigment analysis, cytology, transcriptomics, and proteomics, especially in the transgenic peach callus system, which achieves overexpression and gene editing, indicated that the aggregation of lycopene or β-carotene drives chromoplast differentiation in peach fruits and calli. Specifically, the aggregation of lycopene and β-carotene leads to remodeling of the thylakoid membrane, and the excess crystalline lycopene and β-carotene activate the SGRL-regulated chlorophyll degradation process, leading to degradation of the photosynthetic system (Fig. 9). These findings expand the current knowledge of peach carotenoid accumulation and provide novel insights into how carotenoid aggregates affect chromoplast synthesis.

## Materials and methods

### Plant materials and treatment

The DXMT peaches (a late-maturing variety in which mature fruits are always green) used in the present study were purchased from the fruit market. Whole fruits were soaked in 0.25% CPTA solution (containing 3% glycerol) and water (containing 3% glycerol, control) for 5 min. After drying, the fruits were sealed in a bag and stored in a constant-temperature incubator at 22 °C in a dark environment. From the 8th day after treatment, samples were collected every 3 days. The fruit peels of the treatment and control groups were isolated for sampling, frozen in liquid nitrogen, and stored at −80°C. The XinBaiHua (XBH) immature fruits were collected according to the fruit development stage and subjected to the same CPTA treatment.

DXMT peaches were also treated with 10 μmol/L or 20 μmol/L norflurazon solution (containing 3% glycerol). The processing and storage methods were the same as those for the CPTA solution.

ZJB (*Prunus persica* L. Batsch) calli were cultured under 2000–2500 lx illumination for 18 h each day and maintained at 25 ± 2 ℃ (Liu et al. 2023).

### Pigment analysis

A 0.2 g sample was extracted with 10 ml of chlorophyll extract solution (absolute ethanol: acetone = 1:2) for 12 h in the dark, and the absorbance of the solution was measured at 645 nm and 663 nm.

Carotenoids were extracted and characterized, with 1.0 g of the fresh sample was used for carotenoid extraction (Wang et al. 2023). Carotenoids were separated and analyzed by HPLC-PAD, and qualitative analysis was performed with standard sample spectral absorption and peak retention time. The peak areas of the corresponding carotenoids were recorded at 285 nm, 350 nm, and 450 nm. Data collection and processing were performed with Empower 3 software.

### Transmission electron microscopy

Peel samples with 11-day-old and 20-day-old calli were fixed, dried, stained, and examined under a transmission electron microscope as previously described (Cao et al. 2012). Observation and image acquisition were performed with an HT7700 (HITACHI) transmission electron microscope (TEM).

### RNA-seq library construction, sequencing, and analysis

Total RNA was extracted via a polysaccharide and polyphenol plant total RNA extraction kit (Tiangen, Beijing, China). Fifteen qualified mRNA libraries (Day 0 peach peel, Day 8 CPTA, Day 8 CK peel, Day 11 CPTA, and Day 11 CK peel, with three biological replicates for each sample) were sequenced via the Illumina NovaSeq 6000 sequencing platform. HISAT2 was used to construct a reference genome index and align paired-end clean reads to the peach reference genome (International Peach Genome et al., 2013). Fragments per kilobase of transcript per million mapped reads (FPKM) values were used for gene expression level quantification. The raw counts of each gene were normalized via DESeq2, and genes with a fold change ≥ 1 and *P* ≤ *0.05* were defined as differentially expressed genes (DEGs). The gene set enrichment analysis (GSEA) tool was used to perform Gene Ontology (GO) and Kyoto Encyclopedia of Genes and Genomes (KEGG) enrichment analyses on the DEGs to predict the main functions and degrees of enrichment of genes in specific metabolic pathways. Some genes in the chlorophyll metabolism pathway and carotenoid metabolism pathway were selected for verification of the transcriptome data.

### Total RNA isolation, cDNA synthesis, and reverse transcription quantitative PCR analysis

RNA extraction and cDNA synthesis were performed as previously described (Wang et al. 2021). The gene-specific primers used for qRT‒PCR analysis are shown in Supplemental Table S1. The qRT‒PCR procedure and calculation of relative gene expression were performed as previously described (Wang et al. 2021).

### Plasmid construction and transformation

The *PSY1* and *PSY2* genes from peach fruit were subsequently cloned and inserted into the *pCambia1300S* vector, and the primers used are listed in Supplemental Table S1. To generate *sgrl* CRISPR/Cas9 knockout mutants, two guide RNA sequences were designed to target the exon of *SGRL* (Table S1) and were subsequently cloned and inserted into the *pYLCRISPRCas9P35S-N* vector (Ma et al. 2015).

Loose peach calli were selected for genetic transformation via a previously described method (Cao et al. 2012; Wang et al. 2024). Positive identifications were made after three rounds of selection on solid media containing 10 mg/L hygromycin. Twenty-day-old calli were used for analysis in this study.

### Phylogenetic analysis

The sequence of AtSGR/SGRL was searched in the peach genome of NCBI (https://www.ncbi.nlm.nih.gov) via the BLASTp method. The amino acid sequences were aligned via DNAMAN software. The phylogenetic tree was constructed via the neighbor‒joining method with bootstrapping (1000 replicates) in MEGA 6.

### Carotenoid-engineered bacterial system

The complete *SGRL* and *sgrl* (early terminated SGRL sequence) gene sequences were cloned and inserted into the *pRSFDuet* vector to generate *pRSF::SGRL* and *pRSF::sgrl* (Table S1), respectively. The vectors were subsequently cotransformed into engineered *E. coli* BL21 with *pAC-LYC* (producing lycopene) and *pAC-BETA* (producing β-carotene). The transformed *E. coli* cells were cultured at 37 °C to an OD_600_≈0.5, induced by the addition of 1 mM isopropyl b-D-thiogalactoside (IPTG), and incubated overnight at 20°C. The final bacterial OD_600_ value was recorded, and the cells were collected for imaging and carotenoid analysis.

### Protein extraction, mass detection, and analysis

The peptides obtained after the total plant protein was digested by trypsin were separated via an ultrahigh-performance liquid phase system and injected into the NSI ion source for ionization, followed by analysis via an Orbitrap Exploris™ 480 (Thermo Fisher Scientific) mass spectrometer. Proteome Discoverer software was used to search the peach protein database (https://www.uniprot.org/), and the protein intensity value was used for protein quantification. Proteins with a fold change > 1.5 and *P* < *0.05* were defined as DEPs. Protein functional enrichment analysis was performed via GO and KEGG analyses. Subcellular localization prediction was performed via WolF Psort software. PPDB (http://ppdb.tc.cornell.edu), Plprot (http://www.plprot.ethz.ch/), and SUBA (http://suba.plantenergy.uwa.edu.au/) were used for plastid protein analysis.

#### Western blot analysis

Total protein extraction and western blot analysis were performed as previously described (Cao et al. 2012), with 30 μg of protein used for immunoblotting. Rabbit polyclonal antibodies (Agrisera, Sweden) against phytoene synthase (PSY), plastoglobulin 35 (PGL35), and two photosynthetic proteins, namely, Lhcb1 (LHCII type I chlorophyll a/b-binding protein) and PsbA protein (D1 protein of photosystem II), were used to detect protein abundance via western blot analysis.

#### Molecular dynamics (MD) simulations

MD simulations of lipid bilayers containing lycopene, β-carotene, and phytoene (Fig. S1) were performed with the GROMACS 2021.5 software package (Abraham et al. 2015). A 10% molar concentration is the maximum solubility of carotenoids reported thus far in molecular dynamics simulation studies of carotenoids (Mostofian et al. 2020). In the present study, data from the last 10 ns of MD, including the number of molecular contacts, interaction energy, and lipid bilayer thickness, were collected for quantification.

See the *SI Appendix and Materials and Methods* for details.

#### Yeast two-hybrid assay

The split-ubiquitin yeast two-hybrid system was performed as described previously (Sun et al. 2022b). The PCR products of the full-length *SGRL*, mutant *sgrl* and *SGRL-CT* genes and the pNXgate32-3HA linear vector were co-transformed into yeast AP5, and the *PSY* and pMetYCgate linear vectors were co-transformed into yeast AP4. Single clones were selected for mating and screened in SD/-Leu-Trp medium. Protein interactions were then analyzed based on the growth of yeast in SD/-Leu-Trp-His-Ura-Ade-Met medium.

#### Data analysis

Student's t test was used to analyze significant differences via SPSS statistical software (SPSS Inc., Chicago, USA). GraphPad Prism software (San Diego, CA, USA) and PhotoShop (Adobe Systems, CA, USA) were used to create images. The data are presented as the means ± SDs of triplicate samples.

## Supplementary Information

Additional file 1: Supplemental Methods Detailed information on Molecular dynamics (MD) simulation. Fig. S1. Molecular structure of lycopene, β-carotene and phytoene. Fig. S2. CPTA treatment cannot cause lycopene accumulation in immature peach fruits. Fig. S3. Transcriptome PCA analysis. Fig. S4. Transcriptome validation. Fig. S5. Transcriptome differential gene analysis. Fig. S6. SGR and SGRL phylogenetic analysis. Fig. S7. Analysis of the interaction between SGRL and PSY proteins. Fig. S8. Proteomic data quality control and differential protein volcano plot. Fig. S9. Thylakoid-related protein expression trends in CPTA/CK and PSY1-OE/ZJB. Fig. S10. Photosynthetic system protein expression trend in CPTA/CK and PSY1-OE/ZJB. Fig. S11. Molecular dynamics simulation of carotenoid molecules in lipid bilayers.

Additional file 2: Table S1. Primers used in the study. Table S2. Summary of transcriptome data quality. Table S3. Analysis of specific proteins in peach fruit and callus.

## Data Availability

The data will be available from the corresponding author upon reasonable request.

## Abbreviations

CBB: Coomassie brilliant blue
CCD: Carotenoid cleavage dioxygenase
CPTA: 2-(4-Chlorophenylthio)-triethylamine hydrochloride
DEG: Differentially expressed gene
DEP: Differentially expressed protein
DGDG: Digalactosyldiacylglycerol
DXMT: Dongxue Mitao
ECM: Engineered cell model
GO: Gene Ontology
HPLC-PAD: High performance liquid chromatography- Photodiode array detector
KEGG: Kyoto encyclopedia of genes and genomes
ko: Knockout
LCYB: Lycopene cyclase
LHCII: Photosystem II subunit
MD: Molecular dynamics
MGDG: Monogalactosyldiacylglycerol
NFZ: Norflurazon
NYC: Chlorophyll b reductase
OE: Overexpression
PCA: Principal components analysis
PDS: Phytoene desaturase
PG: Phosphatidylglycerol
PGs: Plastoglobules
PSY: Phytoene synthase
qRT-PCR: Quantitative real-time polymerase chain reaction
SGR: Stay-green
SGRL: Stay-green-like
SQDG: Sulfoquinovosyldiacylglycerol
TEM: Transmission electron microscope
XBH: XinBaiHua
