# Supplementary material for: Carotenoid aggregates negatively impact chlorophyll levels and disrupt chloroplast development in peaches
Source: Mol Hortic. 2026 Apr 10;6:30. doi: 10.1186/s43897-025-00213-8 (PMC13067727; doi:10.1186/s43897-025-00213-8)
Supplement: Supplementary file 1 — Additional file 1: Supplemental Methods Detailed information on Molecular dynamics (MD) simulation. Fig. S1. Molecular structure of lycopene, β-carotene and phytoene. Fig. S2. CPTA treatment cannot cause lycopene accumulation in immature peach fruits. Fig. S3. Transcriptome PCA analysis. Fig. S4. Transcriptome validation. Fig. S5. Transcriptome differential gene analysis. Fig. S6. SGR and SGRL phylogenetic analysis. Fig. S7. Analysis of the interaction between SGRL and PSY proteins. Fig. S8. Proteomic data quality control and differential protein volcano plot. Fig. S9. Thylakoid-related protein expression trends in CPTA/CK and PSY1-OE/ZJB. Fig. S10. Photosynthetic system protein expression trend in CPTA/CK and PSY1-OE/ZJB. Fig. S11. Molecular dynamics simulation of carotenoid molecules in lipid bilayers. [file 43897_2025_213_MOESM1_ESM.docx]

**Supplemental Methods.**

**Detailed information on Molecular dynamics (MD) simulations**

Four lipids (PG, SQDG, DGDG and MGDG) and two carotenoids (lycopene and β-carotene) were geometrically optimized by Gaussian 16 with density functional theory B3LYP/def2-SVP level and DFT-D3 dispersion correction. Ambertools21 and ACPYPE were used to construct the general AMBER force field 2 (GAFF2) parameters, Multiwfn was used to fit the restrained electrostatic potential (RESP) charge.

Initial structure of lipid bilayer containing 60 PG, 60 SQDG, 120 DGDG and 160 MGDG was constructed by genmixmem program. 10×10×11 nm3 cubic boxes were established with prebalanced bilayer and different type of carotenoids (40 lycopene or β-carotene, which is inside of bilayer) packed by PACKMOL program, and these systems were separately solvated in TIP3P water, then 0.15 M NaCl were added to kept electrically neutral. Energy minimization was performed by using the steepest descent algorithm with a force tolerance of 500 kJ mol-1 nm-1. In all the three directions, periodic boundary conditions were imposed. Then these systems were relaxed for five times of 1 ns under NPT ensemble and position restraints with a constant of 1000, 400, 200, 100 and 40 kJ mol-1 nm-2 in three directions were performed on heavy atoms of lipids and carotenoids, respectively.

After completing the above steps, 100 ns NPT MD simulation was performed. Pressure was maintained at 1 bar by the Parrinello-Rahman barostat in a semiisotropic manner (xy and z directions) and temperature was maintained at 310 K by the V-rescal thermostat. The LINCS algorithm was performed for constrain bond lengths of hydrogen atoms. Lennard-Jones interactions were calculated within a cutoff of 1.2 nm, and electrostatic interactions beyond 1.2 nm were treated with particle-mesh Ewald (PME) method with a grid spacing of 0.16 nm. All MD simulation results were visualized by UCSF ChimeraX.

**Supplemental Figure:**


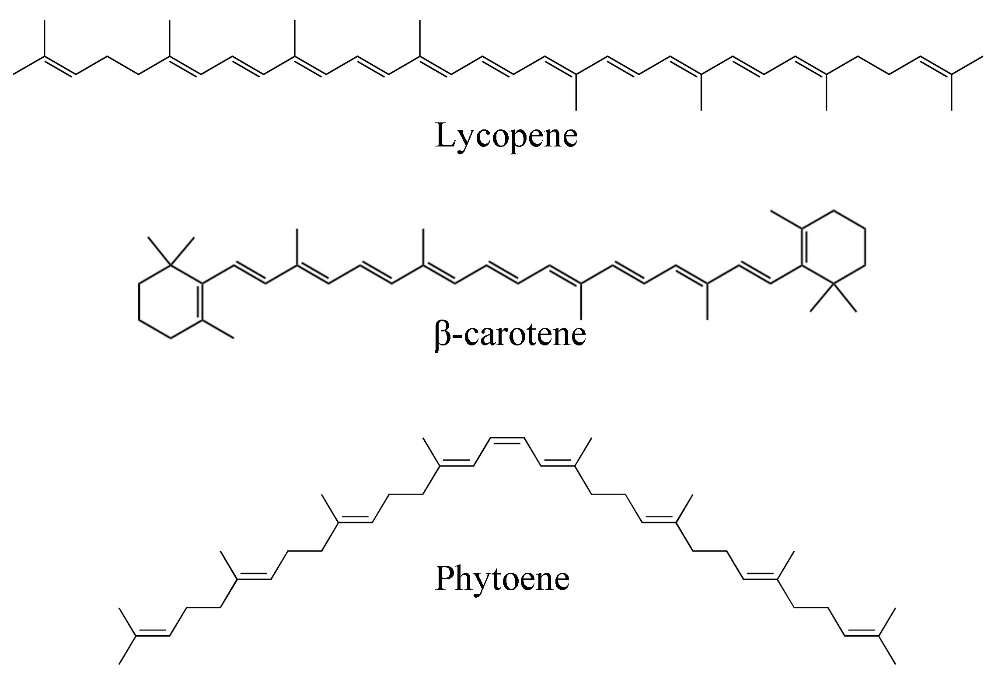


**Supplemental Figure S1** Molecular structures of lycopene, β-carotene, and phytoene used in molecular dynamics simulations.


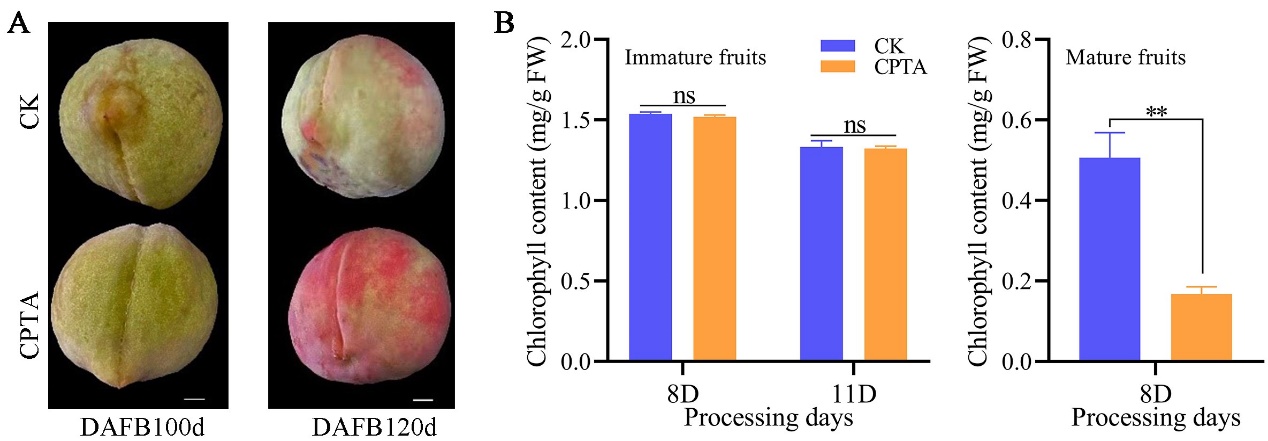


**Supplemental Figure S2** **CPTA treatment cannot cause lycopene accumulation in immature peach fruits.** A: Fruit color after CPTA treatment. (The fruit development period of BeiJing WanMi 'BJWM' is 160 days, and the fruit development period of XinBaiHua 'XBH' is 127 days) (scale bar, 1 cm). DAFB, Days after full bloom. B: Chlorophyll content in pericarp of fruits at different developmental stages (immature and mature stages) after CPTA treatment. FW, fresh weight.


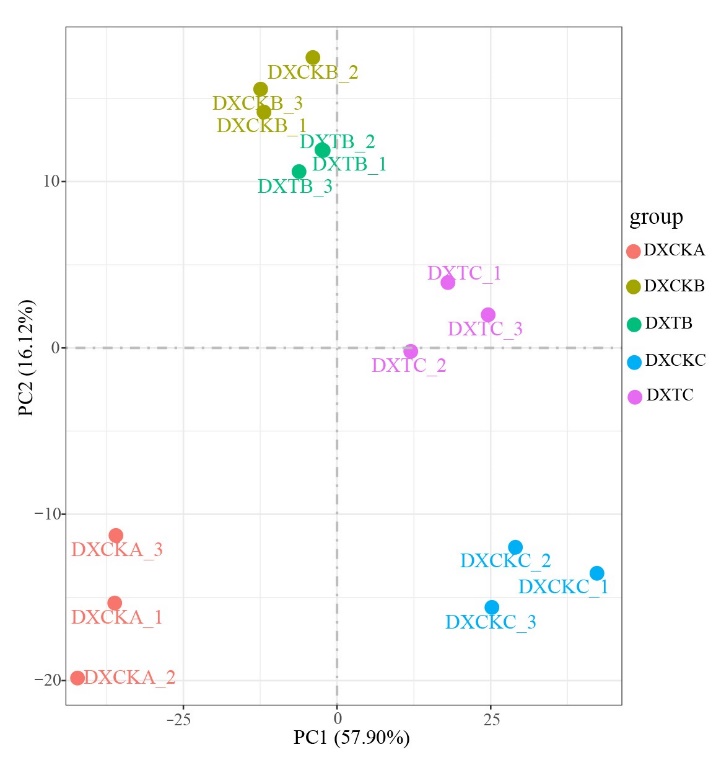


**Supplemental Figure S3 Transcriptome PCA analysis.** PCA analysis was performed on genes identified in 3 biological replicates of CPTA-treatment peels at different periods.


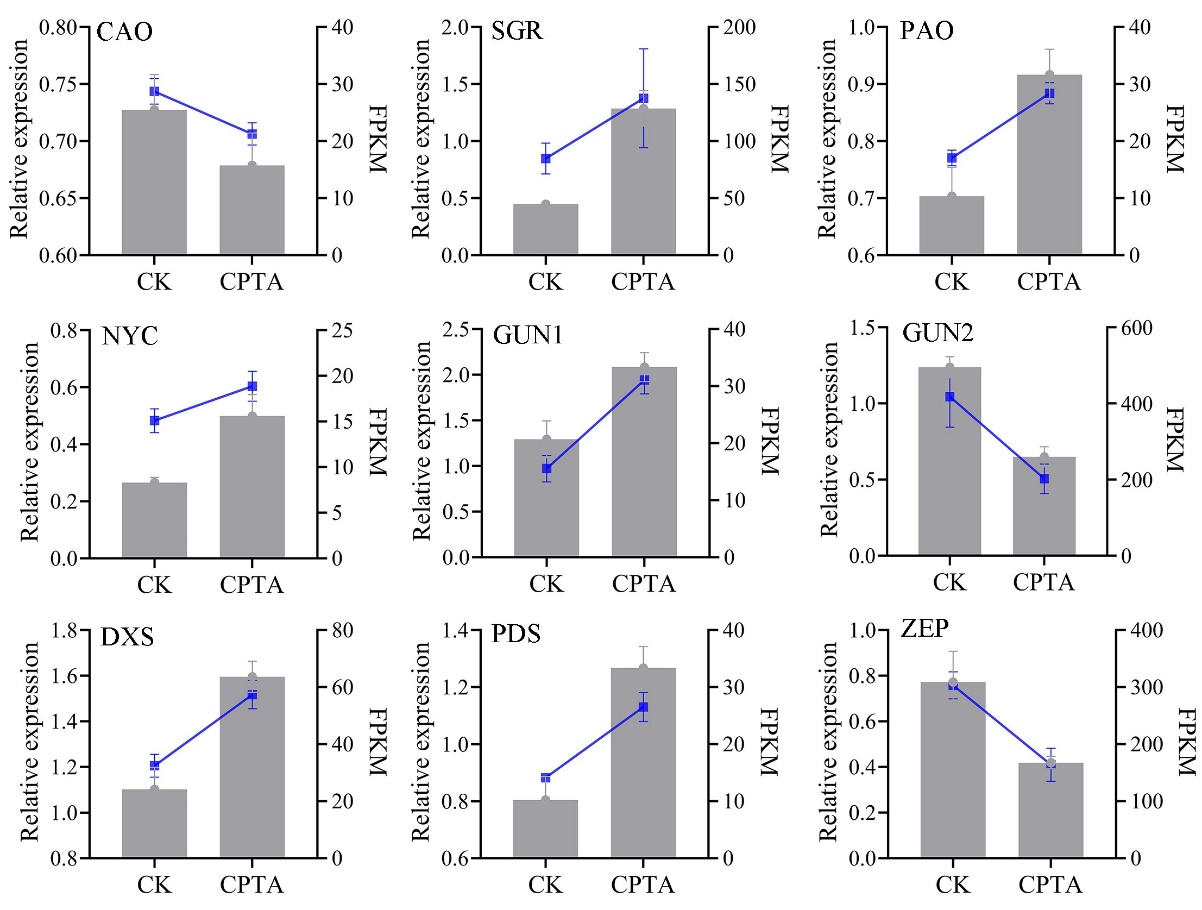


**Supplemental Figure S4 Transcriptome validation.** Expression of genes in the chlorophyll metabolism and carotenoid metabolism pathway measured by RNA-seq (FPKM, histogram) and qRT-PCR (Relative expression, line chart) in 11^th^ day samples. NOTE: CAO, Chlorophyllide a oxygenase; SGR, Stay-green; PAO, Pheophorbide a monooxygenase; NYC, Non-yellow coloring; GUN1, Genomes uncoupled 1; GUN2, Genomes uncoupled 2; DXS, 1-Deoxyxylulose-5-phosphate synthase; PDS, Phytoene desaturase; ZEP, Zeaxanthin epoxidase.


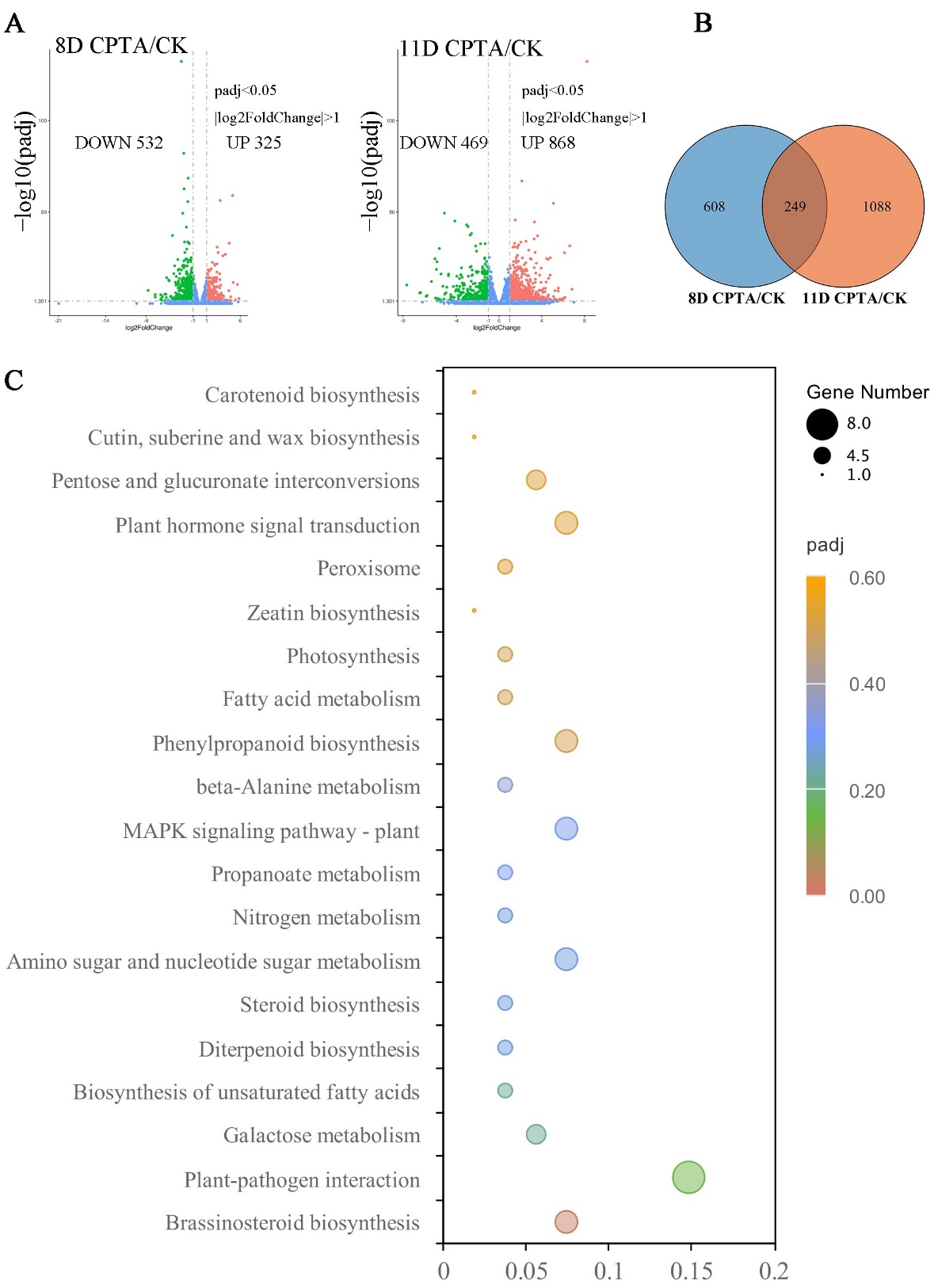


**Supplemental Figure S5** **Transcriptome differential gene analysis.** A: DEGs volcano plots of 8^th^ day and 11^th^ day CPTA-treatment and control. B: Venn diagram of 8^th^ day and 11^th^ day CPTA-treatment and control D: KEGG enrichment map of 11^th^ day CPTA-treatment and control. C: KEGG diagram of 249 differential proteins treated with CPTA for 8^th^ day and 11^th^ day.


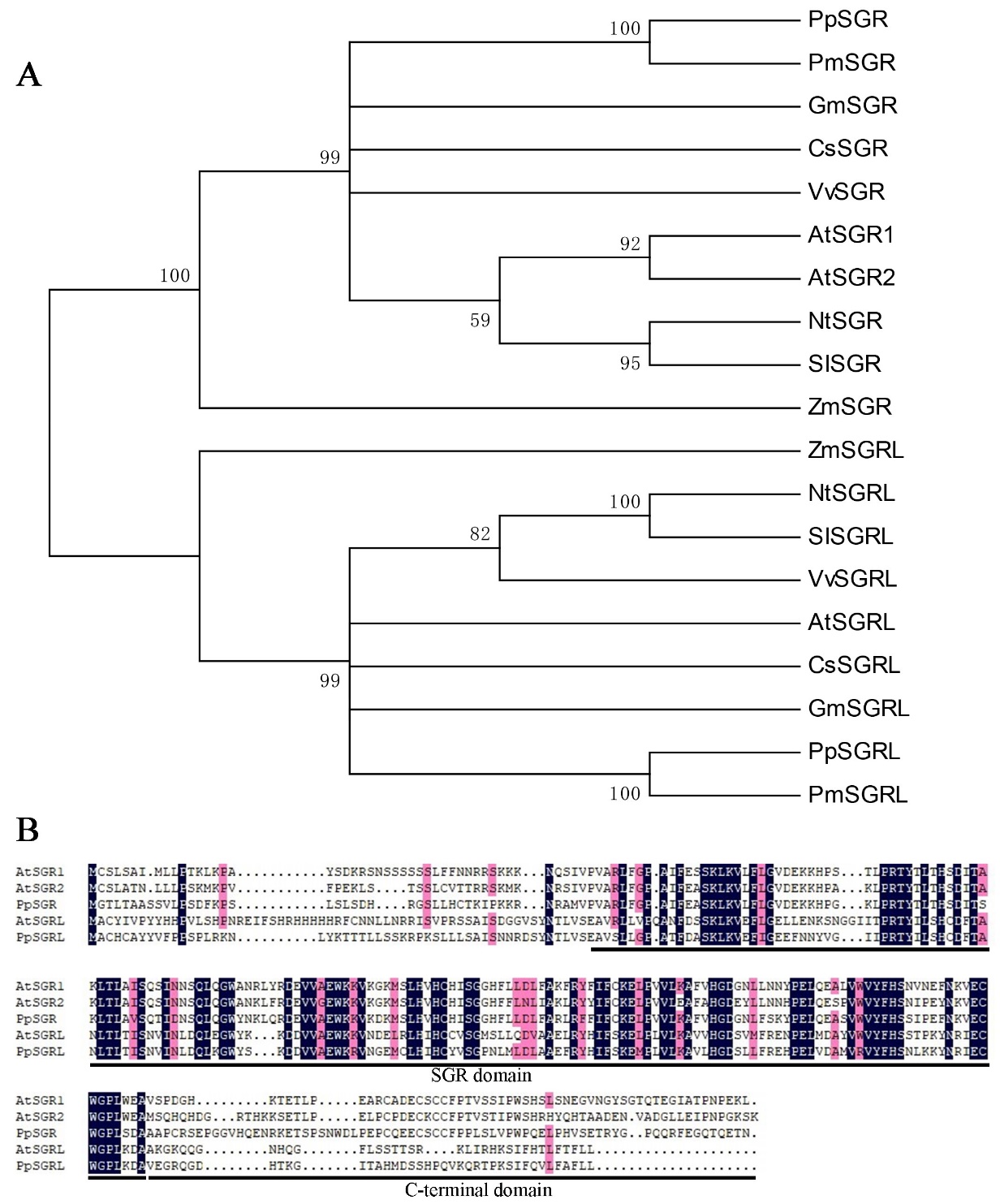


**Supplemental Figure S6** **SGR and SGRL phylogenetic analysis.** A: SGR and SGRL evolutionary analysis. Pm: *Prunus mume*, Pp: *Prunus persica*, Gm: *Glycine max*, At: *Arabidopsis thaliana*, Cs: *Citrus × sinensis*, Vv: *Vitis vinifera*, Nt: *Nicotiana tabacum*, Sl: *Solanum lycopersicum*. B: Amino acid sequence analysis of SGR and SGRL. Contains the SGR domain and the C-terminal domain.


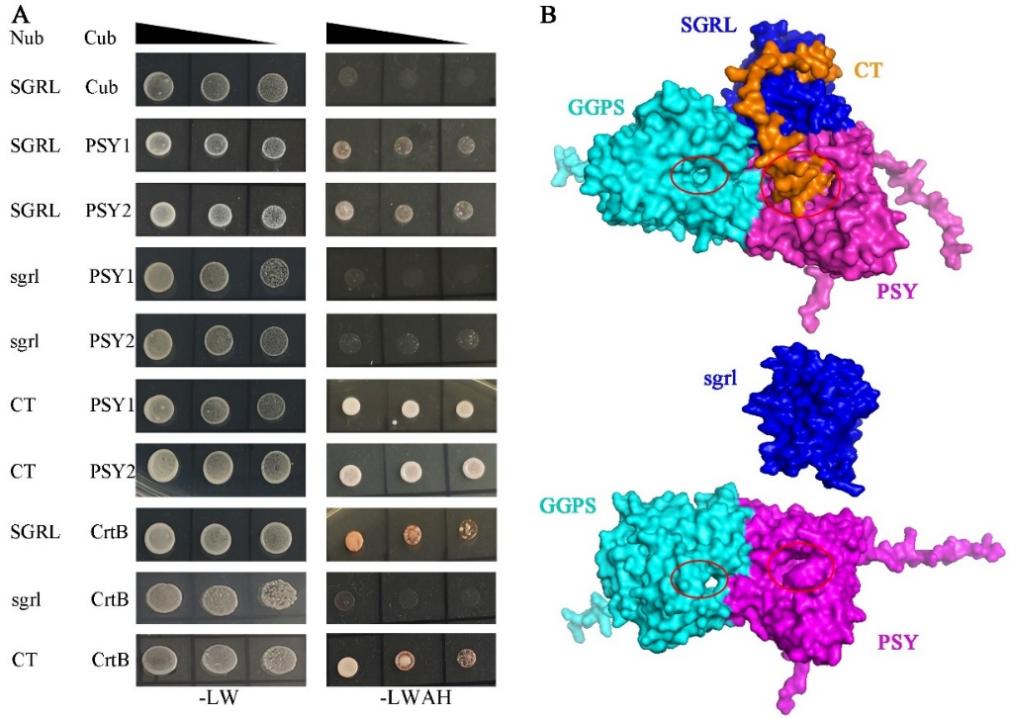


**Supplemental Figure S7 Analysis of the interaction between SGRL and PSY proteins.** A: Y2H interaction verification. SGRL with ubiquitinated N-terminus (Nub) was combined with PSY1/CrtB with ubiquitinated C-terminus (Cub) or empty vector and co-transformed into yeast. After a series of 10-fold dilutions, they were spotted into medium with double deficiency (-LW) or quadruple deficiency containing 200 mM methionine (-LWAH+M) for observation and analysis. B: Molecular docking analysis. The C-terminal domain of SGRL occupies the PSY substrate pocket, resulting in reduced PSY enzymatic activity. The red circle indicates the substrate pocket.


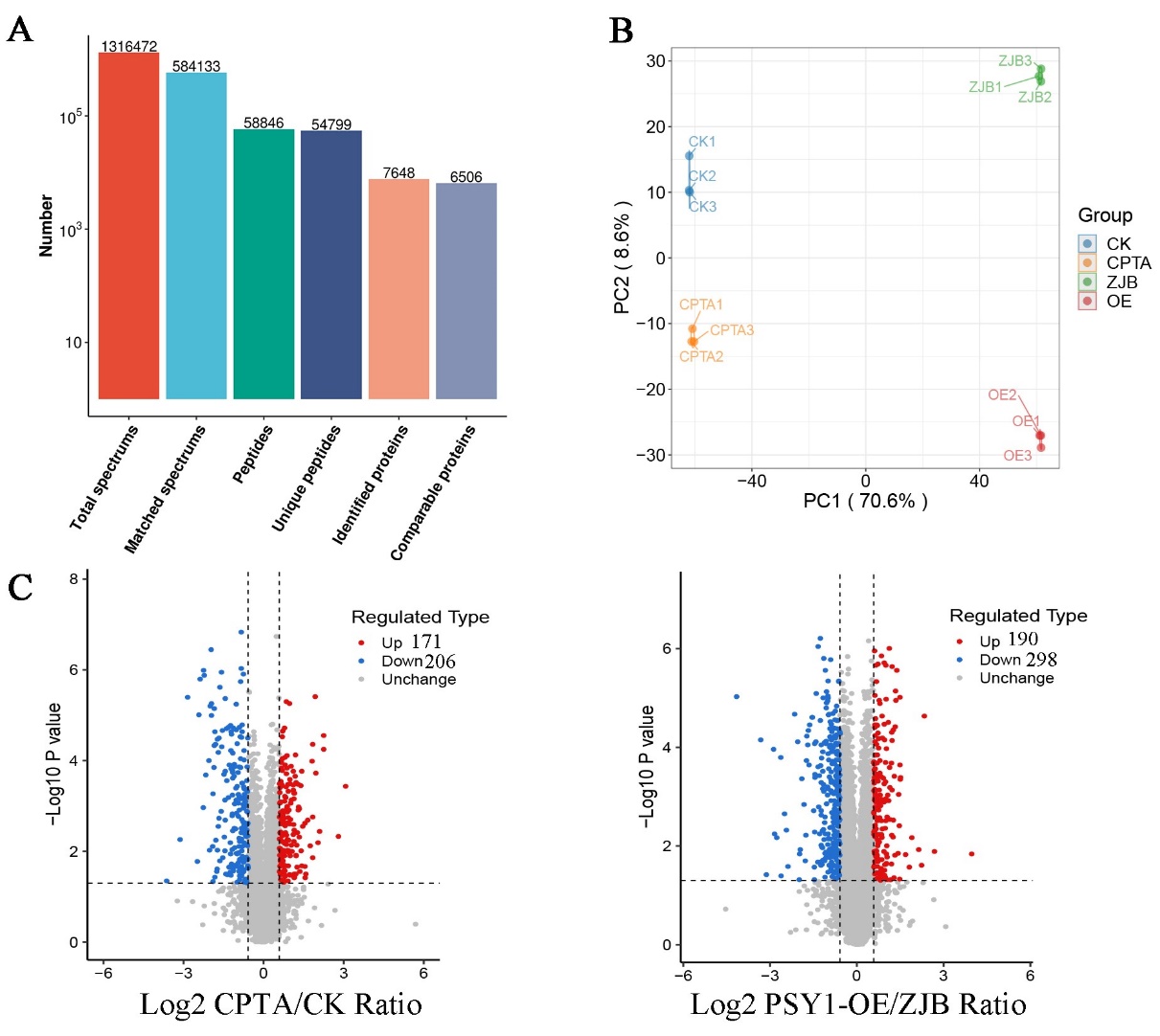


**Supplemental Figure S8** **Proteomic data quality control and differential protein volcano plot.** A: Peptides and proteins identified by mass spectrometry in CPTA-treated peel and *PSY1*-overexpressing callus. B: PCA analysis of total proteins identified in 3 biological replicates of CPTA-treated peel and *PSY1*-overexpressing callus lines. C: Volcano plot of differential proteins. Numbers are shown as protein quantities.


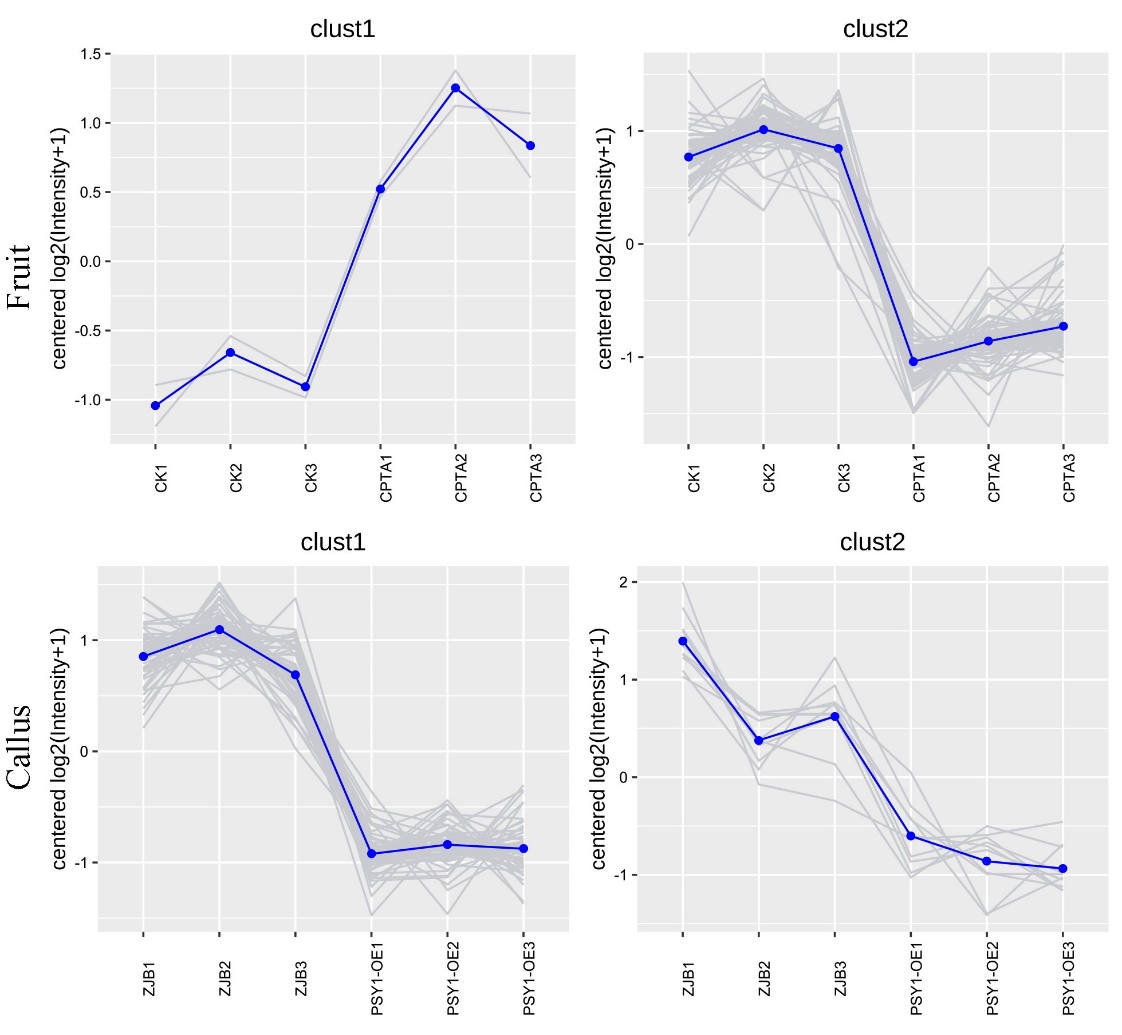


**Supplemental Figure S9** **Thylakoid-related protein expression trends in CPTA/CK and PSY1-OE/ZJB.**


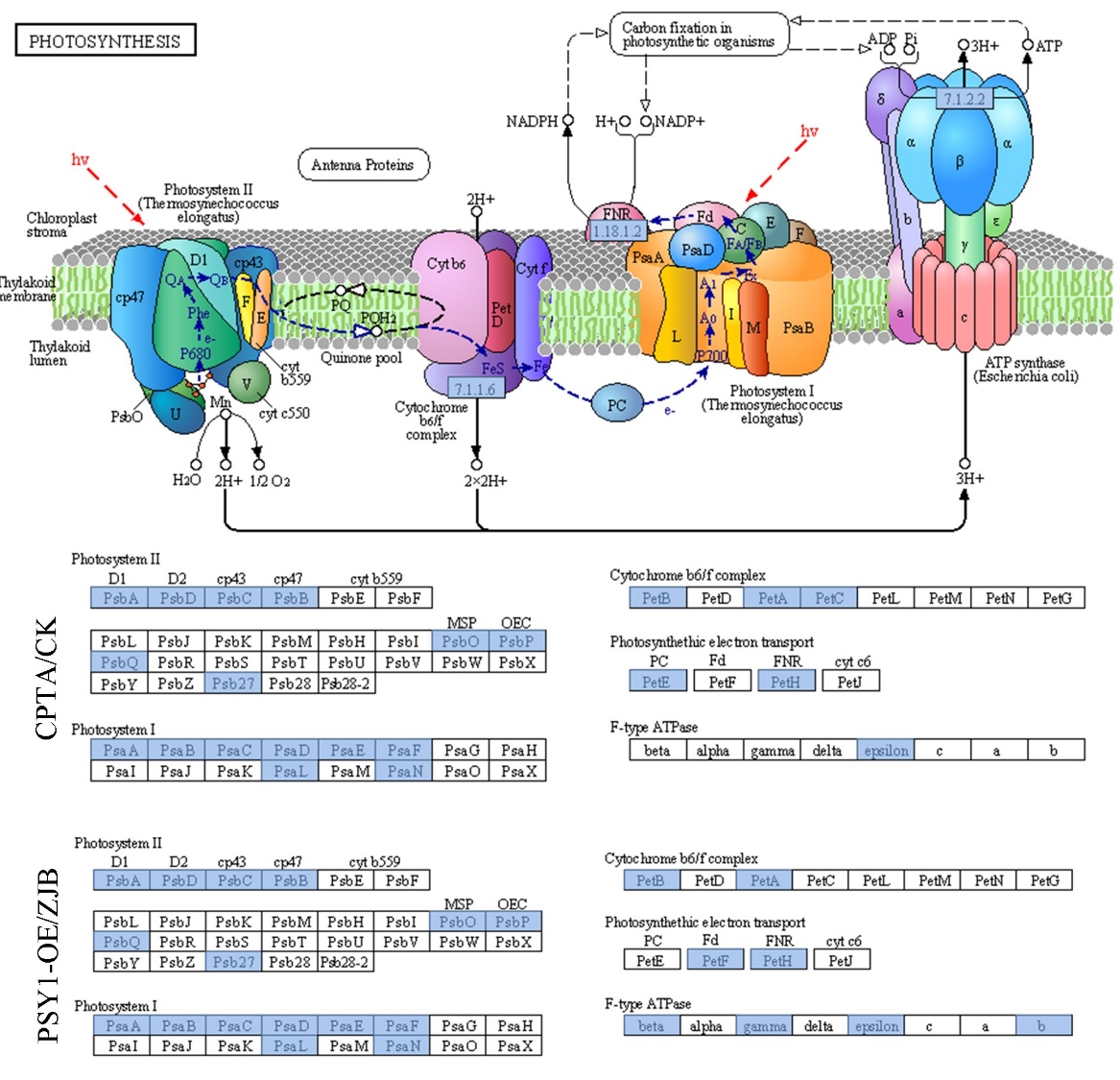


**Supplemental Figure S10** Photosynthetic system protein expression trend in CPTA/CK and PSY1-OE/ZJB. (Blue indicates decreased protein expression).


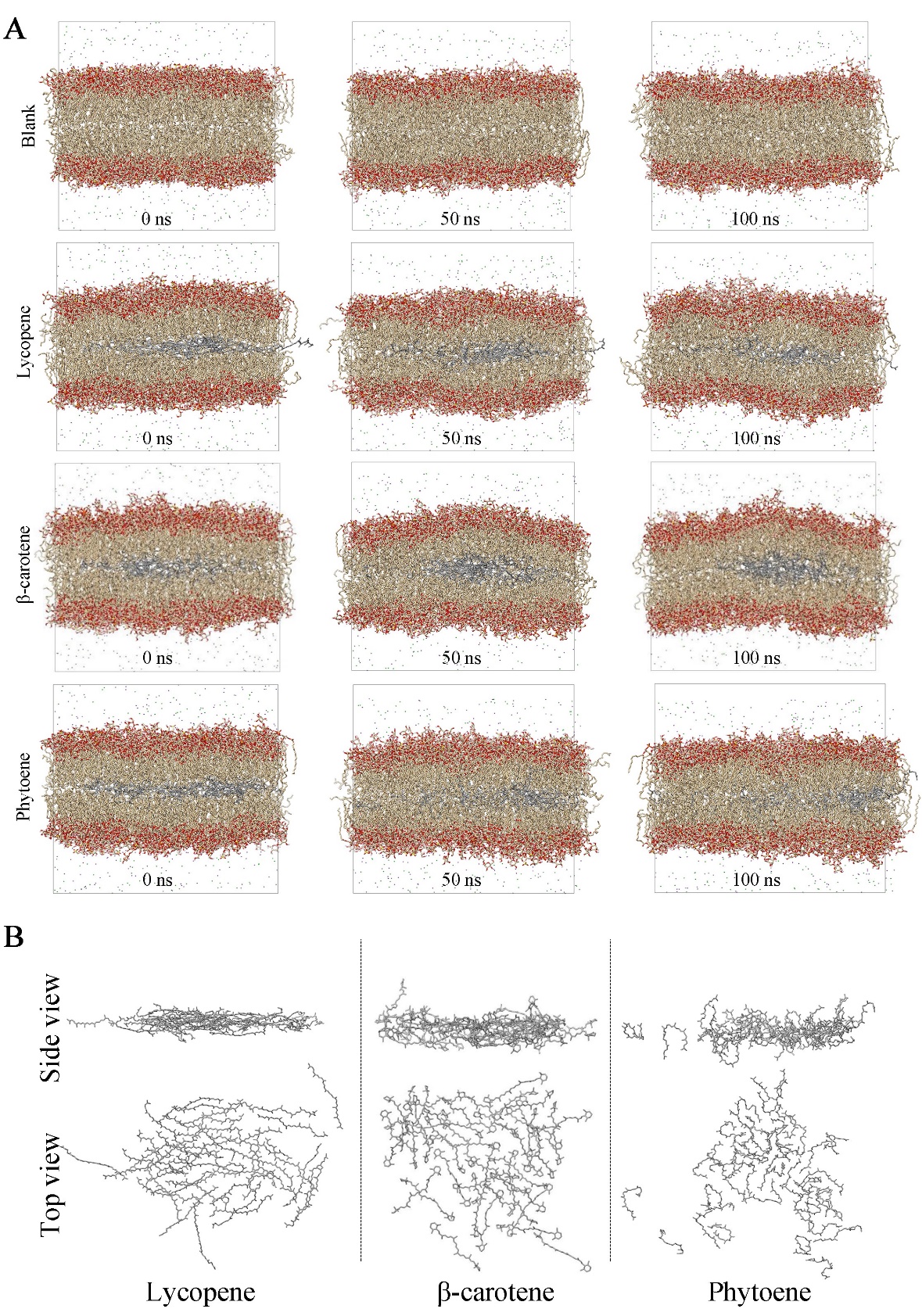


**Supplemental Figure S11** **Molecular dynamics simulation of carotenoid molecules in lipid bilayers.** A: Snapshots of the system at different times during the molecular dynamics simulation (Blank, lycopene, β-carotene, phytoene). B: The final aggregation form of carotenoid molecules. The top is a side view, and the bottom is a top view.

**Supplemental Table**

**Supplemental Table S1**

Primers used in the study.

| Gene names | Forward primers | Reverse primers |
| --- | --- | --- |
| *Rps28*(qRT) | GCATGCTGTGGTTGTGAAAGTT | CCGATTCTGGTCATCCAAAAAC |
| *CAO*(qRT) | TGAAAGGAGGGTTTCGGGTG | GAGCCCGCCAATCACAGTAT |
| *NYC*(qRT) | ATCGTGTGGTTGTCGCTTCT | CAGTTTCTGCACATCGCCAG |
| *CLH*(qRT) | GGTTCTGGTTTGGGTGAGGT | TTCCCTCTGATCCCCTTGGT |
| *PAO*(qRT) | ACCACAAACCACAGACCCAG | CCCAGAAGCTGAAATGGGGT |
| *HCAR*(qRT) | CCTGAACCTGCCCCAAAGTT | AGGTATCCACAATCTTCTTTGCAT |
| *RCCR*(qRT) | TTTACAGACTCCACCACCGC | CATGAGGTTCCTGTGTGGGG |
| *PPH*(qRT) | TTCTGCCTGGTTTTGGGGTT | TTCGTCTCTGGCACACCTTC |
| *SGRL*(qRT) | TCCAGCAACATTCGACGCTT | AACCACGTCATCCTTGCTGT |
| *SGR*(qRT) | ATTGCAGGAGGCTTCGGTTT | TCTTCTTGGCATGGCTCTGG |
| *DXS*(qRT) | GATTGCTTTGGCACTGGTCA | CCCCAAGCCTGCTGAGATAGT |
| *PSY1*(qRT) | GCTTTAGATAGGTGGGAGTC | CTTTGAATGGCTGAATGTC |
| *PSY2*(qRT) | CCCAGAATCAAAGGCTTCAA | ACACTCTTCCTCTCCTGGCA |
| *PDS*(qRT) | GGACAAACAAGGGTGGCTGA | CTTCAAGGTTTTTCGTCACCG |
| *ZEP*(qRT) | ATCCTTGCTCGTGCTGTTG | CCTTCATAACGCTGTCCATT |
| *GUN1*(qRT) | GTTGTGCTTGAAGGGAAGCG | CTATGTTTGCCCCACCCTGT |
| *GUN2*(qRT) | TCGCAATCCCAGTCCCTTTT | CTGTGGTGGCAGAAACAACC |
| *PSY1*(cloning) | atgtctgtagcattactatgggtggt | ctagacttttgatgtcaaggaatcagt |
| *PSY2*(cloning) | atgtcaggtgttcttctttgggtggt | tcatctcgacaccaactgcttagt |
| *1300S-PSY1* | TTTCGCGAGCTCGGTACCatgtctgtagcattactatgggtggt | CTGCAGGTCGACTCTAGActagacttttgatgtcaaggaatcagt |
| *1300S-PSY2* | TTTCGCGAGCTCGGTACCatgtcaggtgttcttctttgggtggt | CTGCAGGTCGACTCTAGAtcatctcgacaccaactgcttagt |
| *koT1-SGRL* | AGCGGTCCCAATCTCATGCgttttagagctagaaat | GCATGAGATTGGGACCGCTCaatcactacttcgtct |
| *koT2-SGRL* | CCTCTCAAGGATGCTGTAGgttttagagctagaaat | CTACAGCATCCTTGAGAGGCaatctcttagtcgact |
| *RSFSGRL* | CCACAGCCAGGATCCGAATTCgatggcctgtcattgtgcttat | GCATTATGCGGCCGCAAGCTTtcaaagaaggaatgcaaata |
| *RSFsgrl* | CCACAGCCAGGATCCGAATTCgatggcctgtcattgtgcttat | GCATTATGCGGCCGCAAGCTTttaagcatccttgagaggc |
| *Y2HSGRL* | acaagtttgtacaaaaaagcaggctctccaaccaccatggaggctgttagtcttttg | tccgccaccaccaaccactttgtacaagaaagctgggtaaagaaggaatgcaaatag |
| *Y2Hsgrl* | acaagtttgtacaaaaaagcaggctctccaaccaccatggaggctgttagtcttttg | tccgccaccaccaaccactttgtacaagaaagctgggtaagcatccttgagaggccc |
| *Y2HSGRL-CT* | acaagtttgtacaaaaaagcaggctctccaaccaccatggtagagggaagacaagggga | tccgccaccaccaaccactttgtacaagaaagctgggtaaagaaggaatgcaaatag |

**Supplemental Table S2**

Summary of transcriptome data quality.

| **sample** | **clean_reads** | **clean_bases** | **Q20** | **Q30** | **total_reads** | **total_map** | **unique_map** |
| --- | --- | --- | --- | --- | --- | --- | --- |
| DXCKA_1 | 39433282 | 5.91G | 97.59 | 93.25 | 39433282 | 37237987(94.43%) | 35749080(90.66%) |
| DXCKA_2 | 49481050 | 7.42G | 97.55 | 93.18 | 49481050 | 45711793(92.38%) | 43783456(88.49%) |
| DXCKA_3 | 46090292 | 6.91G | 97.56 | 93.15 | 46090292 | 42835391(92.94%) | 40976121(88.9%) |
| DXCKB_1 | 46470958 | 6.97G | 97.52 | 92.99 | 46470958 | 43407719(93.41%) | 41951369(90.27%) |
| DXCKB_2 | 41166526 | 6.17G | 97.31 | 92.64 | 41166526 | 37672048(91.51%) | 36518837(88.71%) |
| DXCKB_3 | 44176698 | 6.63G | 97.29 | 92.62 | 44176698 | 41289658(93.46%) | 39998773(90.54%) |
| DXTB_1 | 42192566 | 6.33G | 97.5 | 93.03 | 42192566 | 39733815(94.17%) | 38450090(91.13%) |
| DXTB_2 | 43948488 | 6.59G | 97.6 | 93.2 | 43948488 | 41592016(94.64%) | 40206090(91.48%) |
| DXTB_3 | 42857512 | 6.43G | 97.28 | 92.6 | 42857512 | 39936647(93.18%) | 38607422(90.08%) |
| DXCKC_1 | 41929174 | 6.29G | 97.36 | 92.68 | 41929174 | 38260430(91.25%) | 37481631(89.39%) |
| DXCKC_2 | 42047658 | 6.31G | 97.51 | 92.97 | 42047658 | 38213983(90.88%) | 37337960(88.8%) |
| DXCKC_3 | 43362342 | 6.5G | 97.44 | 92.9 | 43362342 | 39673497(91.49%) | 38652645(89.14%) |
| DXTC_1 | 42663036 | 6.4G | 97.39 | 92.72 | 42663036 | 37733139(88.44%) | 36859382(86.4%) |
| DXTC_2 | 41113502 | 6.17G | 97.51 | 92.99 | 41113502 | 38616517(93.93%) | 37694173(91.68%) |
| DXTC_3 | 44356796 | 6.65G | 98.07 | 94.55 | 44356796 | 39677611(89.45%) | 38737166(87.33%) |
